# Activation and enhancement of caerulomycin A biosynthesis in marine-derived *Actinoalloteichus* sp. AHMU CJ021 by combinatorial genome mining strategies

**DOI:** 10.1186/s12934-020-01418-w

**Published:** 2020-08-06

**Authors:** Yunchang Xie, Jiawen Chen, Bo Wang, Tai Chen, Junyu Chen, Yuan Zhang, Xiaoying Liu, Qi Chen

**Affiliations:** 1 Key Laboratory of Functional Small Organic Molecule Ministry of Education and Jiangxi’s Key Laboratory of Green Chemistry, Key Laboratory of Protection and Utilization of Subtropic Plant Resources of Jiangxi Province, College of Life Sciences, Jiangxi Normal University, Nanchang, 330022 China; 2 School of Life Sciences, Anhui Medical University, Hefei, 230032 China; 3 Guangdong Provincial Key Laboratory of Genome Read and Write, Shenzhen Engineering Laboratory for Innovative Molecular Diagnostics, Guangdong Provincial Academician Workstation of BGI Synthetic Genomics, BGI-Shenzhen, Beishan Industrial Zone, Shenzhen, 518083 China; 4 China National GeneBank, BGI-Shenzhen, Jinsha Road, Shenzhen, 518120 China

**Keywords:** Caerulomycin A, Genome mining, Combinatorial strategies, Ribosome engineering, Strain improvement, Marine-derived *Actinoalloteichus*

## Abstract

**Background:**

Activation of silent biosynthetic gene clusters (BGCs) in marine-derived actinomycete strains is a feasible strategy to discover bioactive natural products. *Actinoalloteichus* sp. AHMU CJ021, isolated from the seashore, was shown to contain an intact but silent caerulomycin A (CRM A) BGC-*cam* in its genome. Thus, a genome mining work was preformed to activate the strain’s production of CRM A, an immunosuppressive drug lead with diverse bioactivities.

**Results:**

To well activate the expression of *cam*, ribosome engineering was adopted to treat the wild type *Actinoalloteichus* sp. AHMU CJ021. The initial mutant strain XC-11G with gentamycin resistance and CRM A production titer of 42.51 ± 4.22 mg/L was selected from all generated mutant strains by gene expression comparison of the essential biosynthetic gene-*camE*. The titer of CRM A production was then improved by two strain breeding methods via UV mutagenesis and cofactor engineering-directed increase of intracellular riboflavin, which finally generated the optimal mutant strain XC-11GUR with a CRM A production titer of 113.91 ± 7.58 mg/L. Subsequently, this titer of strain XC-11GUR was improved to 618.61 ± 16.29 mg/L through medium optimization together with further adjustment derived from response surface methodology. In terms of this 14.6 folds increase in the titer of CRM A compared to the initial value, strain XC-GUR could be a well alternative strain for CRM A development.

**Conclusions:**

Our results had constructed an ideal CRM A producer. More importantly, our efforts also had demonstrated the effectiveness of abovementioned combinatorial strategies, which is applicable to the genome mining of bioactive natural products from abundant actinomycetes strains.

## Background

Actinomycetes, the acknowledged producers of bioactive secondary metabolites, are historically important bioresources in the discovery and development of pharmaceutical molecules [1–5]. In particular, marine actinomycetes thriving under the extreme environmental conditions are different from their terrestrial counterparts, which enables marine-derived strains to acquire excellent characteristics for the biosynthesis of numerous bioactive secondary metabolites [6, 7]. Thus, marine actinomycetes are becoming the emerging resources for exploring novel drug leads [8, 9].

However, the production of bioactive secondary metabolites in marine actinomycetes is always hampered due to unavailable expression of their biosynthetic gene clusters (BGCs) under laboratory conditions [8, 10, 11]. To unlock the biosynthesis potential of these strains, the innovative genome mining strategy-ribosome engineering is adopted to activate the expression of essential biosynthetic genes of cryptic secondary metabolites in strain’s genome [12–18]. Ribosome engineering can usually introduce mutations into the bacterial RNA polymerase β-subunit or ribosomal protein S12 by using rifampicin or streptomycin, respectively [14, 16, 17]. These induced mutations can modulate gene expression to activate biosynthesis of secondary metabolites, leading to discover bioactive natural products from marine actinomycetes [19, 20].

Caerulomycin A (CRM A), a new marine immunosuppressive agent with diverse bioactivities, was isolated from the marine-derived actinomycete *Actinoalloteichus* sp. 2216-6 [21]. The biosynthesis of this marine drug lead is initiated by the formation of a core 2,2′-dipyridine skeleton, which is catalyzed by a unique hybrid polyketide-nonribosomal peptide (PKS-NRPS) assembly line [22–26]. Then, the 2,2′-dipyridine skeleton is finally converted into the CRM A by a series of post-modification reactions, including amino hydrolysis, oxime formation and methylation [23, 27–30]. CRM A can induce the generation of regulatory T cells to avoid T cell responses and change the function of B cells, which significantly inhibits the Mixed Lymphocyte Reaction (MLR) [31, 32]. These prominent bioactivities enable CRM A to be a promising immunosuppressive drug lead, and it has been used in prolonging the survival of allogeneic skin grafts [33].

Given the pharmaceutical prospect of CRM A, screening more original marine strains and solving the yield limitation problems could be an efficient approach in its future development. *Actinoalloteichus* sp. AHMU CJ021 was a new offshore sediment-derived actinomycetes strain. The subsequent sequencing analysis confirmed an intact BGC responsible for CRM A biosynthesis was located in the strain’s genome. However, this strain could not produce CRM A because its BGC was always silent in laboratory fermentations.

Thus, to activate the biosynthesis of CRM A in *Actinoalloteichus* sp. AHMU CJ021, a gentamycin-resistant mutant strain XC-11G was obtained by gene expression-directed ribosome engineering. This strain, unlike the rifamycin or streptomycin-induced mutant strains, did not have definite mutation sites but could produce CRM A with an initial titer of 42.51 ± 4.22 mg/L. Then this titer was rapidly enhanced to 618.61 ± 16.29 mg/L (≈ 14.6 folds) in a following mutant strain XC-11GUR by the subsequent combinatorial methods: UV mutagenesis, cofactor engineering-directed riboflavin supplement optimization, medium optimization integrated with response surface methodology-oriented adjustment. In conclusion, our study had finally woken up the silent gene cluster *cam* and significantly enhanced the CRM A production in *Actinoalloteichus* sp. AHMU CJ021. This reconstruction of new CRM A producer also established the combinatorial strategies composed of strain breeding methods (ribosome engineering, UV mutagenesis and genetic engineering) and medium optimization, which could be effectively utilized in genome mining bioactive natural products and efficient producing strains.

## Results

### Genomic analysis of the CRM A biosynthetic potential in *Actinoalloteichus* sp. AHMU CJ021

**Fig. 1:**
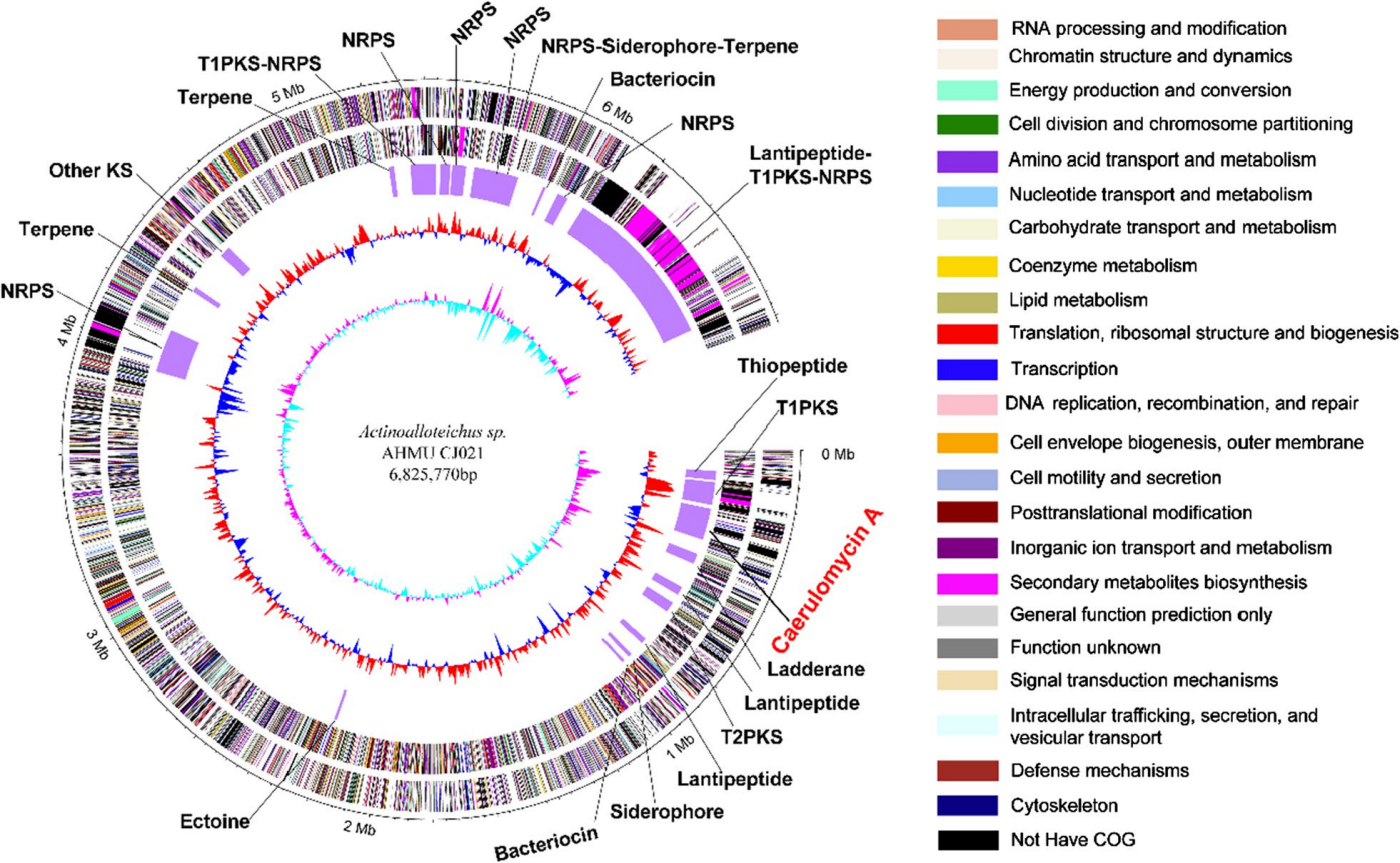
Genome map of *Actinoalloteichus* sp. AHMU CJ021. The genomic information is represented as five circles that depict the following (from outer to inner): (1) and (2) CDS in the sense strand and antisense strand, respectively, with genes coloured according to their COG functional annotations (listed on right); (3) locations of predictive secondary metabolite clusters; (4) G + C content (0–100%) as plotted using a sliding window, as the derivation from the average GC content of the entire sequence. The red peaks and blue peaks indicate the G + C content of the entire sequence in the sense strand and antisense strand, respectively. (5) GC skew (G − C/G + C), as plotted as the deviation from the average GC skew (0–50%) of the entire sequence. The pink peaks and blue-green peaks indicate the G + C content of the entire sequence in the sense strand and antisense strand, respectively

**Fig. 2:**
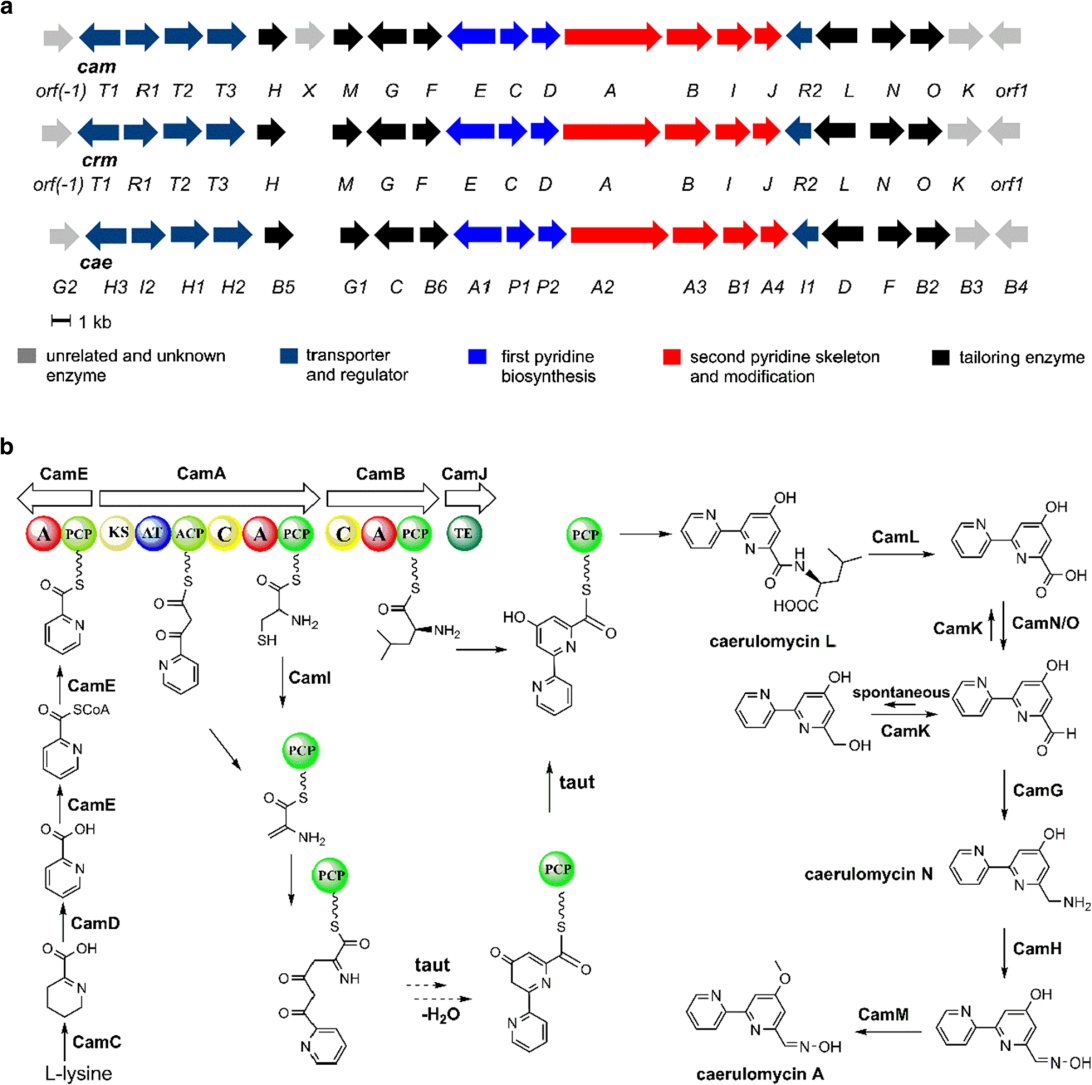
**a** Genetic organization of the CRM A biosynthetic gene clusters in *Actinoalloteichus* sp. AHMU CJ021 (*cam*), *A. cyanogriseus* WH1-2216-6 (*crm*) and *Actinoalloteichus cyanogriseus* NRRL B-2194 (*cae*). **b** The proposed model for caerulomycin A biosynthesis in *Actinoalloteichus* sp. AHMU CJ021. A: adenylation domain; C: condensation; ACP: acyl carrier protein; PCP: peptidyl carrier protein; AT: acyltransferase; KS: β-ketoacyl-ACP synthase; TE: thioesterase

**Fig. 3:**
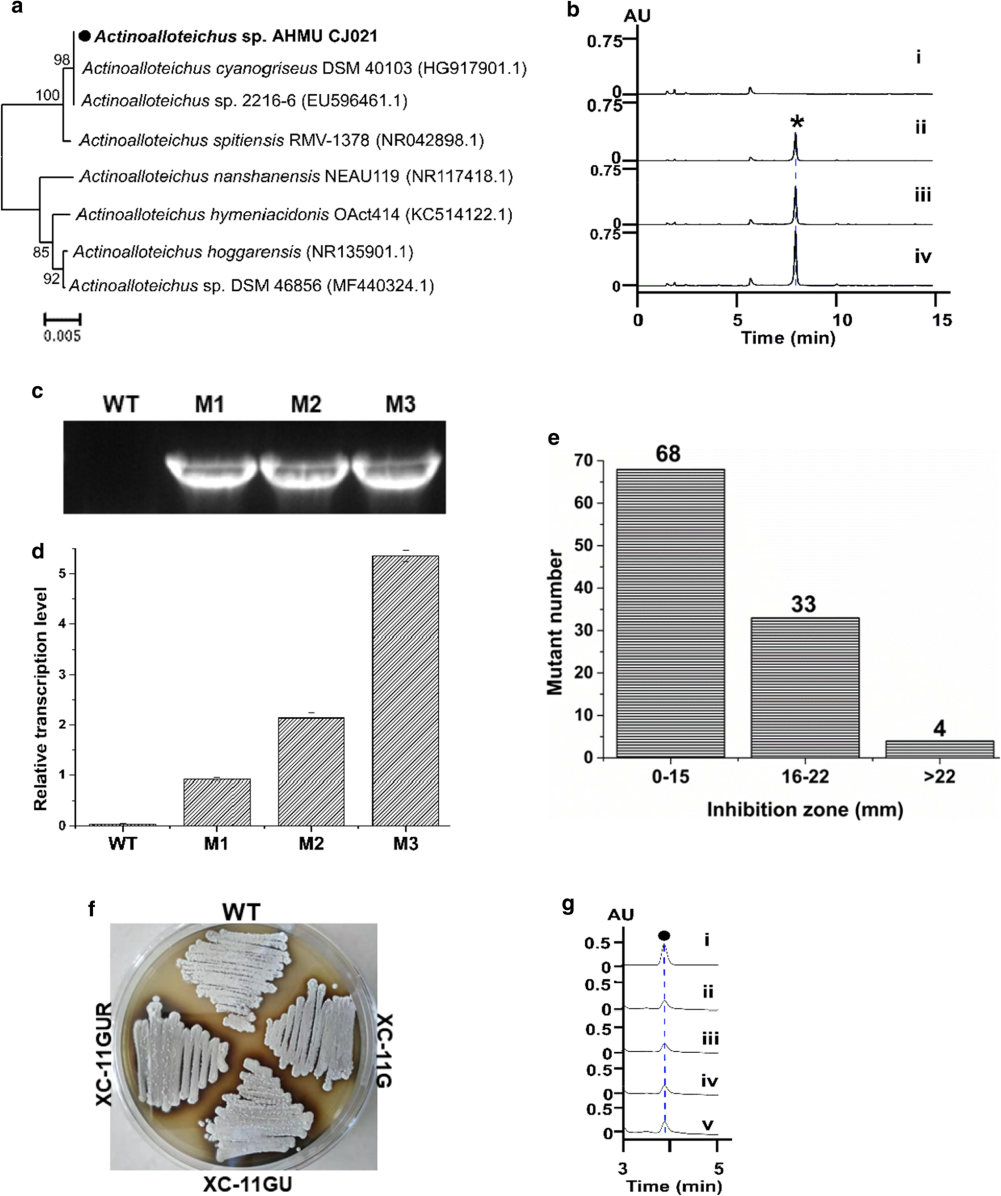
**a** Phylogenetic analysis of *Actinoalloteichus* sp. AHMU CJ021. Numbers at the nodes represent bootstrap percentages obtained from 1000 replicates. The scale bar (0.005) represents nucleotide substitutions per site. Sequence alignment was performed with ClustalW. A phylogenetic tree was constructed with MEGA 7.0 software using neighbor-joining algorithm based on 16S rDNA gene sequences; **b** HPLC analyses of CRM A production (asterisk) in *Actinoalloteichus* sp. AHMU CJ021 wild type and mutant strains. (i) *Actinoalloteichus* sp. AHMU CJ021 wild type; (ii–iv) XC-11G, XC-11GU and XC-11GUR respectively; Transcriptional analysis, **c** RT-PCR assay and **d** qPCR assay, determined *camE* expression in the *Actinoalloteichus* sp. AHMU CJ021wild type strain (**WT**) and three mutants: XC-16R (**M1**), XC-14G (**M2**) and XC-11G (**M3**); **e** The bioassay comparison and screening of mutants generated by UV mutagenesis, the asterisk indicates the inhibition zone level of the original mutant XC-11G; the number of mutants belonging to each range is indicated up the column; **f** The cultured morphology of the *Actinoalloteichus* sp. AHMU CJ021 wild type strain and three mutants on M-ISP2 medium; **g** HPLC analyses for riboflavin (black solid circle) biosynthesis in the *Actinoalloteichus* sp. AHMU CJ021 wild type and mutant strains. (i) authentic standard of riboflavin; (ii) *Actinoalloteichus* sp. AHMU CJ021 wild type; (iii–v) XC-11G, XC-11GU and XC-11GUR, respectively

*Actinoalloteichus* sp. AHMU CJ021 was isolated from the marine sediment. Considering the unique survival environment and secondary metabolite production potential of this strain, the whole genome scanning was adopted, and a 6,825,770 bp genome (NCBI accession number: CP025990) with an average of 72.31% G + C content was obtained (Fig. 1 and Additional file 1: Tables S1 and S2). Subsequent secondary metabolite biosynthesis potential assessments revealed that approximately 22 BGCs were distributed in the strain genome (Fig. 1 and Additional file 1: Table S3). Among these BGCs, the core region of cluster 3, named *cam* (37.9 kb length, from 245,751 bp to 283,656 bp), shows 95% high similarity to two well-identified CRM A BGCs: *crm* (*Actinoalloteichus cyanogriseus* WH1-2216-6) and *cae* (*Actinoalloteichus cyanogriseus* NRRL B-2194) [23, 26] (Fig. 2). The *cam* contains 20 ORFs including essential dipyridine ring biosynthetic genes and backbone post-modification genes [23, 26, 28–30] (Fig. 2 and Additional file 1: Table S4), which indicates that *Actinoalloteichus* sp. AHMU CJ021 can biosynthesize CRM A with the same classic mechanism as that of the two aforementioned phylogenetically identified homologous strains (Fig. 3a and Additional file 1: Fig. S1).

### Activation of CRM A biosynthesis by gene expression-directed ribosome engineering

Given the high similarity in all three BGCs, *Actinoalloteichus* sp. AHMU CJ021 was fermented to produce CRM A by inoculating the strain into previously used media (Additional file 1: Table S5) or other laboratory media. Unexpectedly, no CRM A was detected from the fermentation extracts (Fig. 3b). Further RT-PCR analysis revealed that the essential biosynthetic gene *camE* responsible for CRM A backbone assembly was not expressed in the abovementioned media (Fig. 3c, d). These gene expression results indicated that *cam* was silent under routine conditions.

To activate the expression of *cam*, *Actinoalloteichus* sp. AHMU CJ021 was subjected to ribosome engineering treatment. Hence, a series of 17 mutants were generated by three antibiotics according to the protocols in the methods section (Additional file 1: Table S6). Then, three *camE*-expressing mutants were further selected by the RT-PCR assay (Fig. 3c). The optimal mutant strain XC-11G with 30 μg/mL gentamicin resistance (2× MIC) and the highest *camE* expression level was finally selected after qPCR analysis (Fig. 3d and Additional file 1: Table S7). Subsequent fermentation and HPLC analysis obviously revealed that strain XC-11G could produce a new compound (Fig. 3b lane ii). To our delight, this compound was identified as CRM A by ^1^H NMR, ^13^C NMR and HR-ESI-MS(+) (Additional file 1: Figs. S2, S3, S4 and Additional file 1: Table S8), which verified that *cam* expression and CRM A biosynthesis were effectively activated. Subsequent measurements further confirmed that the strain XC-11G produced CRM A with a titer of 42.51 ± 4.22 mg/L (Additional file 1: Table S7).

The genetic characterization of *rps12* (NCBI accession number: WP_016698050.1), *rpl6* (NCBI accession number: WP_026419518.1) and 16S rDNA (NCBI accession number: CP025990; 1,052,752–1,054,270) from XC-11G revealed that no clear mutation site was located, which was consistent with previously reported cases [34–37]. Additionally, the genetic stability of XC-11G in CRM A biosynthesis was then validated by successive fermentation comparisons among three generations from XC-11G (Additional file 1: Fig. S5a).

### Enhancement of CRM A production by UV mutagenesis

To improve CRM A production efficiently, the UV mutagenesis (traditional high mutation rate method) was adopted to treat the strain XC-11G. After UV irradiation, 105 mutants (over 99.99% lethal rate) were recovered for the following tube-scale fermentation and bioactivity assays of the corresponding extracts. Through comparison of the inhibition zones between UV mutant strains and the preliminary XC-G11 (inhibition zone approximately 16–22 mm), 4 mutants with improved antibacterial bioactivity (inhibition zone > 22 mm) were screened for subsequent fermentation validation (Fig. 3e). The mutant strain XC-11GU was selected among these 4 mutant strains for its highest CRM A yield titer of 78.62 ± 3.55 mg/L, with an approximately 85% increase compared to that of the preliminary XC-11G (Fig. 3b lane iii and Additional file 1: Table S9). Furthermore, the stability of this improvement in CRM A biosynthesis of XC-11GU was validated by the same fermentation process of evaluating its three successive generations (Additional file 1: Fig. S5b).

### Further enhancement of CRM A production by optimizing intracellular riboflavin supplement

Enhancing the cofactor level by engineering its biosynthetic process could drive metabolic flux to improve the biosynthesis of target metabolites [38]. This new metabolic engineering strategy named as cofactor engineering has been well applied to microbial second metabolites development [39–41]. Previous study had revealed that the biosynthesis of CRM A required several essential flavoenzymes, including CamD, which completed the formation of the picolinic acid precursor; CamH, which catalyzed the formation of the oxime group, and CamK, which maintained the substrate recycling process [23, 28, 29]. Thus, enhancing the intracellular riboflavin supplement could facilitate the formation of these flavoenzymes, which may accelerate CRM A production in turn. As is well-known, the riboflavin biosynthesis is controlled by the catalytic efficiency of type II guanosine triphosphate (GTP) cyclohydrolase (GCH II) [42, 43]. Accordingly, in situ overexpression of this rate-limiting GCH II encoded gene could improve intracellular riboflavin biosynthesis [42], which may provide increased amounts of flavin cofactor into the secondary metabolite biosynthetic pathway. This method has been well utilized in *Nocardiopsis flavescens* CGMCC 4.5723, another marine-derived rare actinomycetes strain, to enhance its production of marinacarboline A [44]. Based on the similarity, the in situ overexpression of *AsribA* (NCBI accession number AUS77540.1), encoding GCH II in *Actinoalloteichus* sp. AHMU CJ021, was implemented to construct a new mutant strain XC-11GUR. Although no obvious growth and morphological changes in XC-11GUR were noted relative to the two aforementioned mutant strains, XC-11G and XC-11GU (Fig. 3f), the riboflavin production titer of XC-11GUR steadily increased to 0.51 ± 0.03 mg/L, with a 35% increase compared with that of the wild type strain and the two other mutant strains (Fig. 3g). Importantly, the expected steady enhancement of CRM A production, with a titer of 113.91 ± 7.58 mg/L and an approximately 45% increase compared with that of the previous strain XC-11GU, was also achieved in the new optimal mutant strain XC-11GUR and its three successive generations (Fig. 3b lane iv and Additional file 1: Fig. S5c).

### Medium optimization for CRM A production

**Fig. 4:**
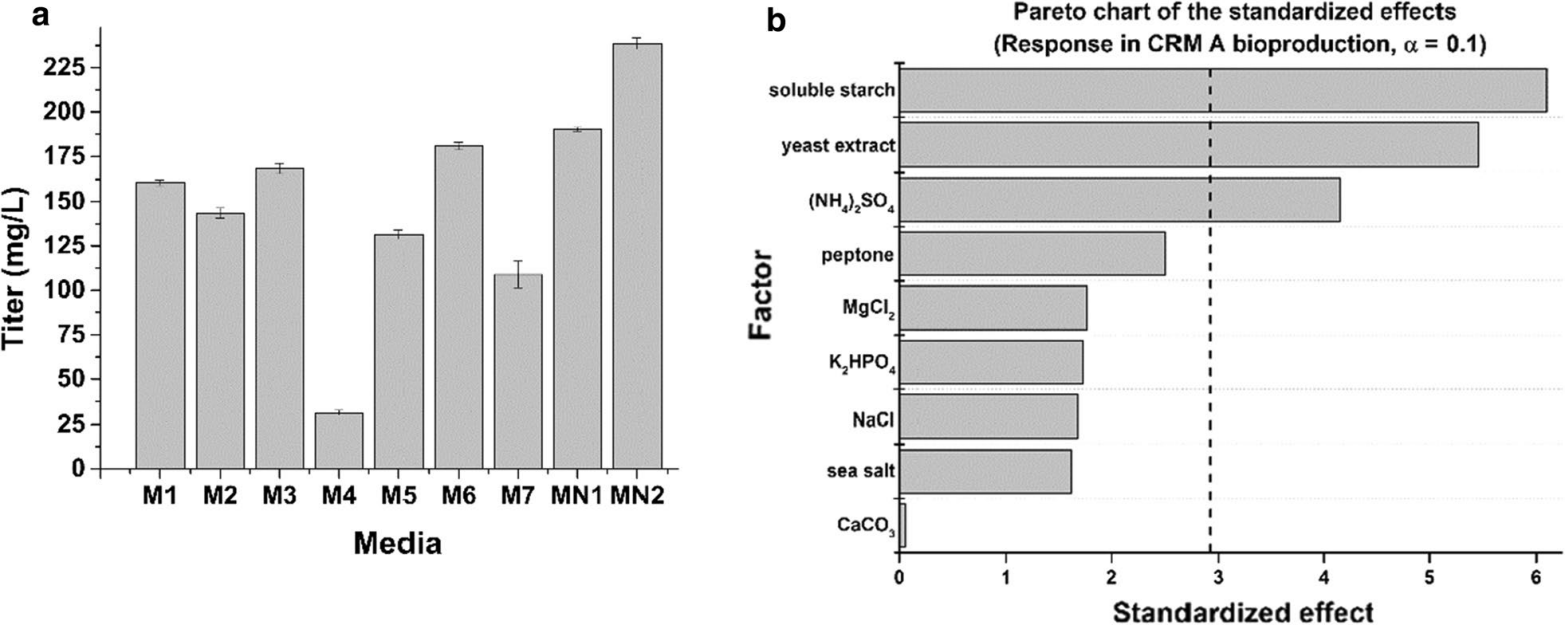
**a** The CRM A production titer on different media (Additional file 1: Table S1); 1–7 represent the Medium 1–Medium 7 (Additional file 1: Table S1); N1 represents the Medium N1 and N2 represents the Medium N2 (Additional file 1: Table S2); **b** Pareto chart displays the standardized effects of each variable and indicates three most significant factors of medium N2 for improving the CRM A production titer. The bars of the diagram that go beyond the vertical dashed line (95% confidence level) correspond to the statistically significant standardized effects

The abovementioned mutant strains were screened and fermented on ISP2 medium, which is convenient for large-batch strain production yield comparison due to its simple but nutritious composition. However, the medium is an essential element in actinomycete secondary metabolites development. Given this important role of the medium, several well-utilized media (Medium 1–7) and two new media (Medium N1–N2) were adopted for CRM A production comparison in mutant strain XC-11GUR fermentation (Additional file 1: Table S5). Among them, the Medium N2, first designed and used in this study, gave rise to the best yield titer of 238.65 ± 3.14 mg/L, which was approximately 1.2–7.6 folds higher than that with the other media (Fig. 4a and Additional file 1: Table S10).

To further improve the fermentation on Medium N2, the significant variables of its in strains’ CRM A production were screened through Plackett-Burman design (PBD). All nine ingredients of medium N2 with addition of the three dummy variables (*X*_*dv1*_–*X*_*dv3*_) were designed as two-level factors for 20 tests (Additional file 1: Tables S11 and S12). After triplicate fermentation comparison, the average CRM A production (Additional file 1: Table S11) was subjected to an effect estimate process (Additional file 1: Table S13). Based on these analyses, three factors, including soluble starch (*X*_*1*_), yeast extract (*X*_*2*_) and (NH_4_)_2_SO_4_ (*X*_*5*_), obviously showed above 95% high confidence level (*p* < 0.05) in the Pareto chart analysis (Fig. 4b). Therefore, these three factors were the critical significant variables of medium N2 optimization to improve CRM A production.

### Further optimization of CRM A production by using response surface methodology

To enhance the CRM A production of strain XC-11GUR on medium N2 to a better level, a Box-Behnken design (BBD) type response surface analysis was employed to determine the optimal concentration of the three variables verified above. After experimental design and fermentation tests (Additional file 1: Tables S14 and S15), the corresponding results and data were subjected to multiple regression analysis. Finally, the equation describing the relationship between CRM A titer (*Y*) and the necessary three variables (soluble starch/*X*_*1*_, yeast extract/*X*_*2*_ and (NH_4_)_2_SO_4_/*X*_*5*_) was confirmed as follow:

$$Y = 572.72 - 80.09X_{1} + 71.51X_{2} - 58.55X_{5} - 59.29X_{1}^{2} - 56.39X_{2}^{2} - 1.8X_{1} X_{2} - 41.4X_{1} X_{5} - 10.19X_{2} X_{5}$$

Additionally, the reliability of the above-obtained model was tested as highly significant by analysis of variance (ANOVA) of CRM A titer to draw high *F*-value and low *p* value (0.00 or < 0.05) in regression coefficient validation (Table 1). Meanwhile, another coefficient estimate showed that most regression coefficients were also highly significant for the low *p*-value (< 0.05). Furthermore, the consistency relevant to two coefficients of determinations: R^2^ (99.66%) and adjusted R^2^ (99.05%), reflected the ideal fit between the observed and predicted responses (Table 2). These agreements once again confirmed present model to be reliable for CRM A production.

**Table 1:** Analysis of variance (ANOVA) for the second-order polynomial model

Source	Degree of freedom	Sum of squares	*F* value	*p* value
Model	9	147,256	163.16	0.000
Linear	3	119,650	397.71	0.000
Quadratic	3	25,786	85.71	0.000
Cross product	3	1821	6.05	0.041
Residual	5	501		
Lack of fit	3	239	0.61	0.672
Pure error	2	263		
Total	14	147,758

**Table 2:** The least-squares fit and coefficient estimate

Variables	Coefficient estimate	Standard error	*t* value	*p* value
Intercept	572.72	5.78	99.06	0.000
*X*_1_	− 80.09	3.54	− 22.62	0.000
*X*_2_	71.51	3.54	20.20	0.000
*X*_5_	− 58.55	3.54	− 16.54	0.000
*X*_1_^2^	− 59.29	5.21	− 11.38	0.000
*X*_2_^2^	− 56.39	5.21	− 10.82	0.000
*X*_5_^2^	− 35.80	5.21	− 6.87	0.001
*X*_1_*X*_2_	− 1.80	5.01	− 0.18	0.864
*X*_1_*X*_5_	− 41.40	5.01	− 4.13	0.009
*X*_2_*X*_5_	− 10.19	5.01	− 1.02	0.356

**Fig. 5:**
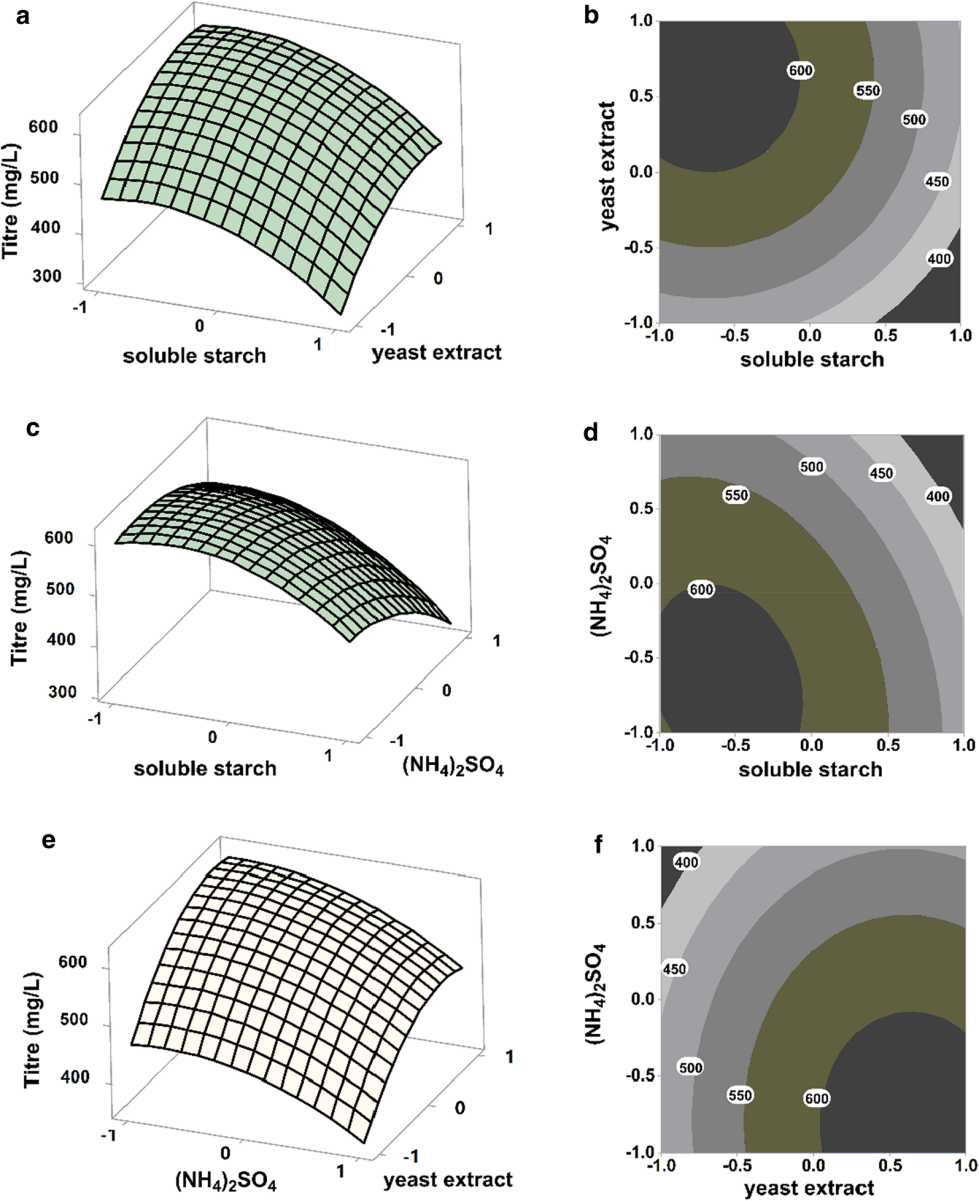
The 3D response surfaces and 2D contour plots for CRM A production in Medium N2. The effect of two variables on CRM A production is presented in each figure, while the other variable is held at zero level. **a**, **b** The effects of soluble starch (*X*_1_) and yeast extract (*X*_2_); **c**, **d** The effects of soluble starch (*X*_1_) and (NH_4_)_2_SO_4_ (*X*_5_); **e**, **f** The effects of yeast extract (*X*_2_) and (NH_4_)_2_SO_4_ (*X*_5_)

**Fig. 6:**
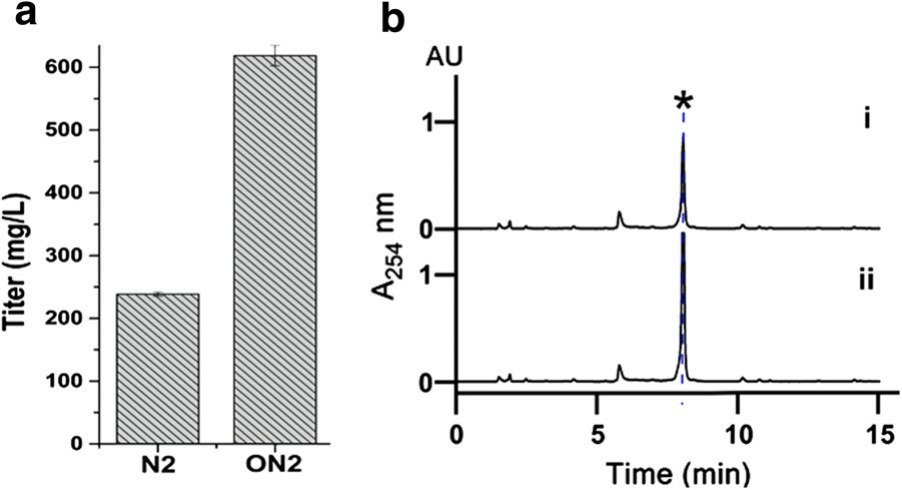
**a** The CRM A production titer of mutant XC-11GUR on Medium N2 (N2) and optimized medium N2 (ON2). **b** The HPLC analysis of CRM A production (asterisk) of mutant XC-11GUR on Medium N2 (i) and Optimized Medium N2 (ii)

The graphical regression equation, displayed on the 3D response surfaces and 2D contour plots (Fig. 5), obviously reflected the optimum selection of three related variables or factors for CRM A titer in the maximal response. Clearly, the CRM A titer improved with increasing concentration of yeast extract/*X*_*2*_ (level  1 ~ 0.7), but with decreasing concentrations of soluble starch/*X*_*1*_ (level 1 ~ − 0.6) and (NH_4_)_2_SO_4_/*X*_*5*_ (level 1 ~ − 0.7). The maximum yield of CRM A based on the optimized ingredient content (*X*_*1*_ level = − 0.556, 10.67 g/L soluble starch; *X*_*2*_ level = 0.677, 3.83 g/L yeast extract; *X*_*3*_ level = − 0.679, 1.00 g/L (NH_4_)_2_SO_4_) was calculated as 639.63 mg/L. To validate this theoretical production, a triplicate confirmatory fermentation with the optimized conditions were performed. As expected, a higher CRM A titer of 618.61 ± 16.29 mg/L was obtained and agreed well with the predicted yield (Fig. 6).

## Discussion

The activation of silent or cryptic secondary metabolite BGCs is an emerging approach in natural product discovery and drug lead development. Antibiotic-induced ribosome engineering is an effective strategy to achieve this goal of activating abundant dormant genes, which in turn fully exploits the potential and valuable bioresources embedded in strain. In the present study, the CRM A biosynthesis potential of marine-derived *Actinoalloteichus* sp. AHMU CJ021 was successfully activated by gentamycin-directed ribosome engineering. Meanwhile, this obtained mutant was also an estimable original strain for subsequent production improvement or further secondary metabolite discovery.

Unlike the well-characterized bioactivities of rifampicin and streptomycin to target bacterial RNA polymerase β-subunit and ribosomal protein S12, respectively, the mechanism of gentamycin-induced mutant is still unclear. The gentamycin-treated *E. coli* could be conferred mutations within 16S-rRNA [45], ribosomal protein S12 [46], or ribosomal protein L6 [47]. In contrast, this mechanism did not reappear in previously identified actinomycete mutants or the current mutant strain XC-11G derived from gentamycin-related ribosome engineering [33, 35, 36]. Although detailed mutation cases could not be clarified in the abovementioned mutant strain XC-11G, the productivity of CRM A is completely activated as a titer of 42.51 ± 4.22 mg/L. This upregulation of gene expression was due to the gentamycin-induced mutation similar to that of antibiotics used in ribosome engineering [12]. Maybe, mutation of the translation apparatus attacked by gentamycin was repaired in subsequent cultivation, which remained upregulated gene expression and drug resistance. These results unambiguously validated the feasibility and efficiency of gentamycin-induced ribosome engineering, even if its detailed mechanism needed to be further investigated.

Significantly, recent progress in genomics and combinatorial biosynthesis research has identified many varied BGCs, providing sufficient authentic information to identify the necessary biosynthetic genes or gene clusters. Thus, the mutation process of ribosome engineering integrated with the expression comparison of essential biosynthetic genes could efficiently single out the desired high-yield and high-quality mutants without time or cost wasted on large-scale fermentation and bioactive comparison. In the present study, we selected *camE* (NCBI accession number: WP_103055331.1), with high similarity to well identified counterparts: *crmE* (NCBI accession number: AFD30957.1) and *caeA1* (NCBI accession number: AFK24513.1) [23, 26], as the molecular marker to rapidly pick out the optimal mutant strain XC-11G from all 17 mutants based on *camE* expression differences. This approach avoided the meaningless and wasteful full-scale production verification or comparison.

Titer improvement is another key issue for production strain development. Traditional UV mutagenesis is a mature technique that induces a high mutation rate into the receptor strains. Engineering the riboflavin biosynthesis by increasing the supplement of the precursor to the flavin coenzyme formation is an efficient pathway that could indirectly activate the secondary metabolite biosynthesis [38–41]. This cofactor engineering type method has been well utilized in another marine actinomycete [44]. A straightforward strategy combining these two methods was applied for further production improvement of the original mutant strain XC-11G to obtain a more desirable mutant strain XC-11GUR, which effectively enhanced the CRM A production titer by approximately 2.7 folds increase. Not surprisingly, these methods combined with previous ribosome engineering could dramatically increase the ratio of positive results to efficiently enhance strain’s CRM A productivity more than the inefficient using of any method alone.

As is known, the medium is another crucial condition for secondary metabolite production. In particular, the proper medium is always an influential factor in the expression of activated BGCs. Considering the important role of medium composition, the optimal mutant XC-11GUR was cultured with nine different media including six previously characterized CRM A production media, two new designed media, and ISP2 used as a convenient choice with simple but nutrient-rich ingredients (Additional file 1: Table S5). The generated CRM A yield varied significantly in these different media, and the optimum medium N2 was screened out, which had glucose (carbon source) removed but ammonium sulfate (inorganic nitrogen source) added. Glucose, a typical fast-acting carbon source, matching organic nitrogen sources is a common basic formula used in secondary metabolite production. However, supplementing some (NH_4_)_2_SO_4_ as an inorganic nitrogen source and removing the commonly used glucose resulted in a significant enhancement in CRM A production. This empirical change provides a new CRM A titer or fermentation condition improvement option and once again confirms the importance of the medium in secondary metabolite production.

To further improve the medium N2, response surface methodology was adopted to determine the significant variables and further evaluate those effects, leading to the establishment of an optimized medium N2 composition (approximately 2.6 folds CRM A titer improvement) containing three critical factors were verified: 10.67 g/L soluble starch, 3.83 g/L yeast extract and 1.00 g/L (NH_4_)_2_SO_4_. Compared with that in the original medium N2, the amount of carbon source was further decreased. More notably, the ratio of yeast extract and peptone (organic nitrogen source) to (NH_4_)_2_SO_4_ (inorganic nitrogen source) was increased from 2:1 (4–2 g/L) to approximately 6:1 (5.83–1 g/L), suggesting that the controlled carbon source feeding and high concentration of an organic nitrogen source coupled with supplementation of a limited inorganic nitrogen source could perfectly meet the requirements of CRM A biosynthesis to improve the CRM A yield in turn.

## Conclusion

Overall, the CRM A production capacity of *Actinoalloteichus* sp. AHMU CJ021 was successfully activated and significantly improved by combining yield-oriented strain improvement via ribosome engineering, gene expression comparison integrated methods, UV mutagenesis, intracellular riboflavin biosynthesis enhancement, and empirical medium optimization coupled with response surface methodology-directed adjustment. Our efforts have obtained a new promising marine-derived CRM A high-yield producer that could be utilized for subsequent rational pathway engineering improvement. Moreover, our work also highlighted the efficacy of combinatorial strategies including empirical strain breeding methods and culture condition improvement approaches in rapid secondary metabolite improvement, which could facilitate discovery and development of natural products.

## Materials and methods

### Strain and culture conditions

*Actinoalloteichus* sp. AHMU CJ021 (the strain has been deposited to the China Center for Type Culture Collection with the number CCTCC M 2018157) was isolated from the ~ 20 cm depth marine sediment on the seashore of Lianyungang, East China Sea. This strain was cultivated on solid ISP2 medium for sporulation. The selected fermentation media were described in Additional file 1: Table S5, which were also used to detect the CRM A production titer. For each fermentation, 50 μL spore suspension (10^7^) of each strain was inoculated into 50 mL liquid fermentation medium supplied with 2% XDA-16 resin in a 250 mL flask, then incubated at 28 °C and 200 rpm for 7 days.

### Whole genome scanning and sequence analysis

The genomic DNA of *Actinoalloteichus* sp. AHMU CJ021 was extracted, sequenced and assembled completely by combining Illumina HiSeq2500 system and PacBio RSII high throughput sequencing technologies (BIOZERON-Shanghai; BGI-Shenzhen). Subsequently, functional genes were predicted by Glimmer and annotated by BlastP (https://blast.ncbi.nlm.nih.gov/Blast.cgi), referring to Swiss-Prot, COG and KEGG databases. The rRNA and tRNA genes were analyzed using RNAmmer and tRNAscan-SE, respectively.

Open reading frames (ORFs) were analyzed with the Frameplot 4.0 program (http://nocardia.nih.go.jp/fp4/) and the Blast program (http://blast.ncbi.nlm.nih.gov/). The PKS-NRPS domains were determined by web-based software (http://nrps.igs.umaryland.edu/nrps/).

The phylogenetic trees of 16S rDNA between *Actinoalloteichus* sp. AHMU CJ021 and homologous strains were constructed with the Molecular Evolutionary Genetics Analysis (MEGA) 7.0 software using the neighbor-joining algorithm.

### Ribosome engineering of *Actinoalloteichus* sp. AHMU CJ021

The minimal inhibition concentration of three commonly used antibiotics, namely, streptomycin, gentamycin, and rifamycin, against *Actinoalloteichus* sp. AHMU CJ021 was determined. Spore suspensions (10^6^–10^7^ spores) of this strain were spread onto ISP2 plates containing different concentrations of the three abovementioned antibiotics (5–100 μg/mL) and cultivated at 28 °C for persistent observation. The following treatments of the strains were carried out by applying the three abovementioned antibiotics at concentrations of 1× MIC–5× MIC on the ISP2 plates to obtain resistant mutant strains.

Genetic characterization of 30S ribosomal protein S12 (NCBI accession number: WP_016698050.1), 50S ribosomal protein L6 (NCBI accession number: WP_026419518.1) and 16S rDNA (NCBI accession number: CP025990; 1,052,752–1,054,270) of the final selected gentamycin-resistant mutant strains was performed by PCR using the primers listed in Additional file 1: Table S16. The amplified oligonucleotides were sequenced to verify the mutations (BGI-Shenzhen).

### Isolation and identification of CRM A from mutant strains

To isolate CRM A from the mutant strains, a two-step fermentation process was adopted. First, a suitable portion of spores from a solid ISP2 medium plate was inoculated into 50 mL ISP2 medium and cultured at 28 °C and 200 rpm for 36 h. Then, this seed culture (50 mL) was transferred into 200 mL ISP2 medium in a 1 L flask and cultured at 28 °C and 200 rpm for an additional 6–7 days. Multiple flasks were used for repeating. Finally, the culture broth was centrifuged and divided into a supernatant and a mycelium cake. The supernatant was extracted by butanone at least two times and evaporated to dryness; the mycelium was extracted with acetone and evaporated to dryness too; Both organic extracts were dissolved in a 1:1 mixture of CHCl_3_/MeOH and mixed with an appropriate amount of silica gel for normal phase silica gel column chromatography, eluted with a successive gradient elution of CHCl_3_/MeOH mixture at 100/0, 98/2, 96/4, 95/5, 93/7, 90/10, 80/20 and 50/50 to yield eight fractions (Fr1–Fr8). The fractions were analyzed in order by HPLC-UV. Fr3–Fr4 were subjected to another normal phase silica gel column chromatography step and eluted with a 100/0-0/100 gradient elution of a ddH_2_O/MeOH mixture to yield thirteen fractions (FrA1–FrA13). FrA5–FrA8 were evaporated to dryness and subjected to a PE/EtOH mixture (7:3, 6:4, 5:5, 4:6, and 3:7) to form five fractions. Each fraction was analyzed by HPLC and dissolved in MeOH and finally purified by semi-preparative HPLC to give the resulting CRM A, as identified by ^1^H, ^13^C NMR and HR-ESI-MS.

Analytical HPLC of CRM A was performed on the Agilent 1260 Infinity System with an Agilent Zorbax SB-C18 column (150 × 4.6 mm, 5 μm) eluted with a linear gradient of 0% to 80% solvent B over 20 min, followed by 80% to 100% solvent B in 30 s, and then eluted with 100% solvent B in 4.5 min, at a flow rate of 1.0 mL/min using UV detection at both 210 nm and 254 nm. Solvent A was composed of 15% CH_3_CN, 85% ddH_2_O, and 0.1% acetic acid, and solvent B composed of 85% CH_3_CN, 15% ddH_2_O, and 0.1% acetic acid.

To measure the strain’s CRM A production titer in the confidence interval, the corresponding quantitative HPLC standard curve was generated by analyzing an authentic CRM A concentration gradient (from 2 to 10 μg, with 2 μg increments). The UV absorption of each analysis was maintained below 1 A unit to ensure appropriate confidence of the generated standard curve. The strain’s fermentation broth (50 mL in a 250 mL flask) was extracted by butanone and concentrated in vacuo to afford the oily residues. These residues were dissolved in 2 mL MeOH. Then a suitable volume of generated methanol solution was subjected to HPLC analysis to acquire the peak area value. Finally, the titer of different strains was calculated, using a computational formula derived from a standard curve and the volume ratio (Additional file 1: Fig. S6a).

### Gene expression analysis of mutant strains

Total RNA of each mutant was extracted using the SV total RNA purification Kit (Promega) and digested by DNase I (Takara). First-strand cDNA synthesis was accomplished using Invitrogen’s SuperScript™ Kit, and second step PCR was carried out under the following conditions: 94 °C for 5 min, 25 cycles of denaturation (94 °C for 25 s), annealing (60 °C for 25 s), and extension (72 °C for 45 s), and a single extension at 72 °C for 10 min. A negative control was accordingly performed in the absence of template to check for DNA contamination after the DNase I digestion required for RNA purification.

Quantitative real-time reverse transcription PCR (qPCR) was performed using the Maxima™ SYBR Green qPCR Mix (MBI) and Applied Biosystem’s 7500 Fast Real-time PCR system. 16S rDNA was used as the internal control. The primers used to analyze the *camE* gene (NCBI accession number: WP_103055331.1) and 16S rDNA are shown in and Additional file 1: Table S17.

### UV mutagenesis and biological assay screening of mutant strains

Diluted spores of selected ribosome engineering mutant strains were put into an uncovered petri dish, which was placed on a magnetic stirrer beneath a UV light on a clean bench. These spores were treated for different irradiation times (5–10 min, with 1 min increments). The preliminary experiments determined that 8 min UV irradiation time confer about 99% lethal rate and was suitable for strains’ mutagenesis operation. After UV irradiation, all strains’ spores were spread onto ISP2 plates for subsequent dark cultivation at 28 °C for 5 days to obtain recovered viable colonies.

The surviving mutant strains were inoculated into test tubes containing 5 mL ISP2 medium and fermented at 28 °C for 3 days. Then, the fermentation product was extracted with butanone three times by the ultrasonic treatment. The obtained fermentation extract was dissolved in 50 μL methanol. Approximately 10 μL liquid extract was dropped onto punched 6 mm filter papers, which were attached on the LB plates inoculated with not yet grown *Escherichia coli* ATCC 25922. The colonies with the largest inhibition zones were screened for subsequent fermentation verification.

### Riboflavin biosynthesis enhancement of mutant strains

The essential riboflavin biosynthetic gene *AsribA* (NCBI accession number AUS77540.1), encoding the rate-limiting type II guanosine triphosphate (GTP) cyclohydrolase (GCH II), was completely synthesized (BGI-Shenzhen) and subsequently cloned into the pCR2.1 vector. The target gene region was digested with *NdeI* and *XbaI* and ligated into the same digested pSET152AKE to obtain the desired plasmid pSET152AKE-*AsribA* [48–50]. This constructed plasmid was transformed into *E. coli* ET12567/pUZ8002 to construct the donor strain *E. coli* ET12567/pUZ8002/pSET152AKE-*AsribA*, which was then cultivated in 100 mL LB with kanamycin (Kan, 50 μg/mL), chloramphenicol (Chlo, 25 μg/mL) and apramycin (Apm 50 μg/mL) at 37 °C and 200 rpm for 4–6 h to an OD of 0.6–0.8. Cells were harvested and washed with liquid LB medium, and resuspended in fresh LB medium (1 mL). The harvested spores of the screened mutant strain were inoculated into 50 mL liquid ISP2 medium, heated for 10 min at 50 °C, cooled to ferment at 28 °C for 6–8 h and then mixed with the above-harvested *E. coli* ET12567/pUZ8002/pSET152AKE-*AsribA* cells. The mixed sample was spread on ISP2 plates supplemented with MgCl_2_ (20 mM). The plates were incubated at 28 °C for 20 h. Then, these plates were covered with sterile water (1 mL) supplemented with 30 μL trimethoprim (TMP, stock solution 50 mg/mL) and 30 μL Kan (stock solution 50 mg/mL). The plates were incubated at 28 °C until exconjugants appeared. The exconjugants were verified by PCR using the amplified primers (asribA-Fr: 5′-*ACG ACG GTG GAG AGC AGG ACG*-3′ and asribA-Re: 5′-*TTA TGC CGT CAC TCC CGT TCC*-3′) to screen the desired gene over-expression mutant.

Analytical HPLC of riboflavin was also performed on Agilent 1260 Infinity System with an Agilent Zorbax SB-C18 column (150 × 4.6 mm, 5 μm) eluted as follow: a linear gradient of 0% to 70% solvent B over 20 min, followed by 70% to 100% solvent B within 15 s, and then eluted with 100% solvent B within 5 min, at a flow rate of 1.0 mL/min with UV detection at 254 nm and 275 nm. Solvent A was composed of 15% CH_3_CN, 85% ddH_2_O, and 0.1% acetic acid, and solvent B was composed of 85% CH_3_CN, 15% ddH_2_O, and 0.1% acetic acid. The standard sample of riboflavin was isolated in a previous study [44].

The quantitative HPLC standard curve for comparing riboflavin production titer was generated by analyzing an authentic riboflavin concentration gradient (from 0.2 μg to 1 μg, with 0.2 μg increments). The UV absorption of each analysis was maintained below 1 A unit to ensure appropriate confidence of the generated standard curve. The strain’s fermentation broth (50 mL in a 250 mL flask) was extracted by butanone and concentrated in vacuo to afford oily residues. These residues were dissolved in 0.5 mL MeOH. Then 10 μL of generated methanol solution were subjected to HPLC analysis. Finally, the titers of different strains were calculated using a computational formula derived from a standard curve and the volume ratio (Additional file 1: Fig. S6b).

### Medium optimization using response surface methodology

The experiments for optimizing the CRM A production medium were designed and evaluated by using the Minitab 17 software. This software can be used in analyzing the statistical experiment data and response surface contour plots to solve the obtained regression equation, which can finally determine the optimal composition of the fermentation medium.

Preliminary identification of significant variables from CRM A production medium was performed by using Plackett–Burman design. The importance of variables was investigated at widely spaced intervals distinguished as low level (− 1) and high level (+ 1). The effects of each variable on CRM A production were directly calculated in Minitab 17 (with the option “Analyze Factorial Design”) by the following equation: E represents the effects of the variable under study, and M^+^ and M^−^ are responses (CRM A production titer) of each trial at which the variable was as its high or low level, respectively; N is the total number of trials. Furthermore, the *t*-values and *p*-values of each variable were calculated in Minitab 17 by analyzing factorial design, which was synchronized with the generation of a Pareto chart.

$${\text{E}} = \left( {\sum {\text{M}}^{ + } - \sum {\text{M}}^{ - } } \right)/{\text{N}}$$

Box-Behnken design could accurately analyze the interaction effects among various ingredients and determine the optimal level of significant factors during CRM A production. In this study, an experimental design consisting of three independent variables at three different levels (− 1, 0, 1) was performed in 15 trials. All trials were repeated in triplicate and the average of CRM A titer was set as response (*Y*). The second order polynomial coefficients were calculated and analyzed using Minitab 17. The general form of the second degree polynomial equation is:

$$Y = \beta_{0 } + \sum \beta_{i } X_{i} + \sum \beta_{ii } X^{2}_{i} + \sum \beta_{ij } X_{i} X_{j}$$

*Y* is the predicted response; *X*_*i*_ and *X*_*j*_ are input variables that influence the response *Y*; *β*_*0*_ is the offset term; *β*_*i*_ is the *i*th linear coefficient; *β*_*ii*_ is the *i*th quadratic coefficient; and *β*_*ij*_ is the *ij*th interaction coefficient. Statistical analysis of the obtained model was performed in ANOVA form. This analysis included the Fisher’s *F*-test (overall model significance), its associated probability *P*(*F*), correlation coefficient R, and determination coefficient R^2^ which measures the goodness of fit of the regression model. For the variables, the quadratic models were displayed as contour plots and response surface curves which were generated using Minitab 17.

## Supplementary information

**Additional file 1: Additional file 1: Table S1.** Genome features of *Actinoalloteichus* sp. AHMU CJ021; **Table S2.** Number of genes associated with the general COG functional categories; **Table S3.** The biosynthetic gene clusters of secondary metabolites in *Actinoalloteichus* sp. AHMU CJ021 analyzed by antiSMASH 5.0; **Table S4.** Deduced functions of the open reading frames (ORFs) indicated in Fig. 2; **Table S5.** Fermentation media used for caerulomycin A (CRM A) production; **Table S6.** The mutants obtained from ribosome engineering experiments; **Table S7.** The CRM A production comparison of three *camE*-expressing mutants; **Table S8.**^1^H NMR and ^13^C NMR spectral data of CRM A in DMSO-*d6*; **Table S9.** The comparison of selected mutants generated from UV mutagenesis; **Table S10.** CRM A production titer of optimal mutant XC-11GUR; **Table S11.** The dose of all factors in medium N2 by using Plackett-Burman Design; **Table S12.** Screening of significant variables for CRM A production in Medium N2 by using Plackett-Burman Design; **Table S13.** The effects of all factors of Medium N2 for CRM A production by using Plackett-Burman Design; **Table S14.** The dose of important factors in response surface analysis; **Table S15.** The design of experiments and response of CRM A production; **Table S16.** The primers used in identification of gentamycin-resistant mutant; **Table S17.** The primers used in gene expression analysis; **Fig. S1.** Phylogenetic tree of *Actinoalloteichus* sp. AHMU CJ021; **Fig. S2.** HR-ESI-MS spectrum of CRM A; **Fig. S3.**^1^H NMR spectrum of CRM A in DMSO-*d6*; **Fig. S4.**^13^C NMR spectrum of CRM A in DMSO-*d6*; **Fig. S5.** CRM A production comparison of different generations of mutants; **Fig. S6.** The quantitative HPLC standard curves.

## Data Availability

All data generated or analyzed during this study are included in this published article and in its additional file.

## Abbreviations

BGC: Biosynthetic gene cluster
WT: Wild type
RT-PCR: Reverse transcription PCR
qPCR: Quantitative real-time PCR
