# Correction to: Activation and enhancement of caerulomycin A biosynthesis in marine-derived *Actinoalloteichus* sp. AHMU CJ021 by combinatorial genome mining strategies

**DOI:** 10.1186/s12934-020-01429-7

**Published:** 2020-09-07

**Authors:** Yunchang Xie, Jiawen Chen, Bo Wang, Tai Chen, Junyu Chen, Yuan Zhang, Xiaoying Liu, Qi Chen

**Affiliations:** 1 Key Laboratory of Functional Small Organic Molecule Ministry of Education and Jiangxi’s Key Laboratory of Green Chemistry, Key Laboratory of Protection and Utilization of Subtropic Plant Resources of Jiangxi Province, School of Life Sciences, Jiangxi Normal University, Nanchang, 330022 China; 2 School of Life Sciences, Anhui Medical University, Hefei, 230032 China; 3 Guangdong Provincial Key Laboratory of Genome Read and Write, Shenzhen Engineering Laboratory for Innovative Molecular Diagnostics, Guangdong Provincial Academician Workstation of BGI Synthetic Genomics, BGI-Shenzhen, Beishan Industrial Zone, Shenzhen, 518083 China; 4 China National GeneBank, BGI-Shenzhen, Jinsha Road, Shenzhen, 518120 China

## Correction to: Microb Cell Fact (2020) 19:159 10.1186/s12934-020-01418-w

Following publication of the original article [1], the authors have flagged that an incorrect version of Fig. 4b has been published.

**Fig. 4:**
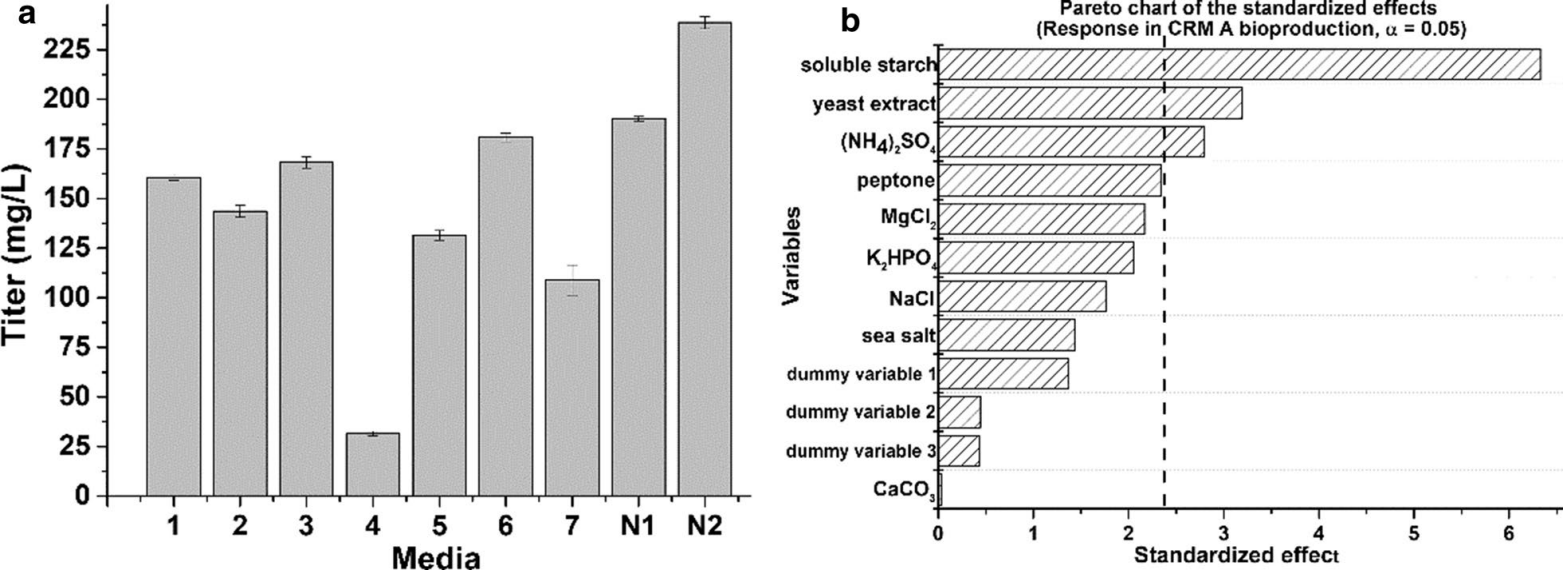
**a** The CRM A production titer on different media (Additional file 1: Table S1); 1–7 represent the Medium 1–Medium 7 (Additional file 1: Table S1); N1 represents the Medium N1 and N2 represents the Medium N2 (Additional file 1: Table S2); **b** Pareto chart displays the standardized effects of each variable and indicates three most significant factors of medium N2 for improving the CRM A production titer. The bars of the diagram that go beyond the vertical dashed line (95% confidence level) correspond to the statistically significant standardized effects

In the published version, the vertical dashed line is incorrectly positioned and the dummy variables are erroneously omitted from the y-axis.

To correct the version provided in the published article, please find (the corrected version of) Fig. 4b in this correction.

The authors apologize for any inconvenience caused.

## Supplementary information

**Additional file 1: Table S1.** Genome features of **Actinoalloteichus** sp. AHMU CJ021; **Table S2.** Number of genes associated with the general COG functional categories; **Table S3.** The biosynthetic gene clusters of secondary metabolites in *Actinoalloteichus* sp. AHMU CJ021 analyzed by antiSMASH 5.0; **Table S4.** Deduced functions of the open reading frames (ORFs) indicated in Fig. 2; **Table S5.** Fermentation media used for caerulomycin A (CRM A) production; **Table S6.** The mutants obtained from ribosome engineering experiments; *Table S7.* The CRM A production comparison of three camE-expressing mutants; **Table S8.** ^1^H NMR and ^13^C NMR spectral data of CRM A in DMSO-d6; **Table S9.** The comparison of selected mutants generated from UV mutagenesis; **Table S10.** CRM A production titer of optimal mutant XC-11GUR; **Table S11.** The dose of all factors in medium N2 by using Plackett-Burman Design; **Table S12.** Screening of significant variables for CRM A production in Medium N2 by using Plackett-Burman Design; **Table S13.** The effects of all factors of Medium N2 for CRM A production by using Plackett-Burman Design; **Table S14.** The dose of important factors in response surface analysis; **Table S15.** The design of experiments and response of CRM A production; **Table S16.** The primers used in identification of gentamycin-resistant mutant; **Table S17.** The primers used in gene expression analysis; **Fig. S1.** Phylogenetic tree of *Actinoalloteichus* sp. AHMU CJ021; **Fig. S2.** HR-ESI-MS spectrum of CRM A; **Fig. S3.** ^1^H NMR spectrum of CRM A in DMSO-d6; **Fig. S4.** ^13^C NMR spectrum of CRM A in DMSO-d6; **Fig. S5.** CRM A production comparison of different generations of mutants; **Fig. S6.** The quantitative HPLC standard curves.
