# Supplementary material for: Activation and enhancement of caerulomycin A biosynthesis in marine-derived Actinoalloteichus sp. AHMU CJ021 by combinatorial genome mining strategies
Source: Microb Cell Fact. 2020 Aug 6;19:159. doi: 10.1186/s12934-020-01418-w (PMC7412835; doi:10.1186/s12934-020-01418-w)
Supplement: Supplementary file 1 — Additional file 1: Additional file 1: Table S1. Genome features of Actinoalloteichus sp. AHMU CJ021; Table S2. Number of genes associated with the general COG functional categories; Table S3. The biosynthetic gene clusters of secondary metabolites in Actinoalloteichus sp. AHMU CJ021 analyzed by antiSMASH 5.0; Table S4. Deduced functions of the open reading frames (ORFs) indicated in Fig. 2; Table S5. Fermentation media used for caerulomycin A (CRM A) production; Table S6. The mutants obtained from ribosome engineering experiments; Table S7. The CRM A production comparison of three camE-expressing mutants; Table S8.1H NMR and 13C NMR spectral data of CRM A in DMSO-d6; Table S9. The comparison of selected mutants generated from UV mutagenesis; Table S10. CRM A production titer of optimal mutant XC-11GUR; Table S11. The dose of all factors in medium N2 by using Plackett-Burman Design; Table S12. Screening of significant variables for CRM A production in Medium N2 by using Plackett-Burman Design; Table S13. The effects of all factors of Medium N2 for CRM A production by using Plackett-Burman Design; Table S14. The dose of important factors in response surface analysis; Table S15. The design of experiments and response of CRM A production; Table S16. The primers used in identification of gentamycin-resistant mutant; Table S17. The primers used in gene expression analysis; Fig. S1. Phylogenetic tree of Actinoalloteichus sp. AHMU CJ021; Fig. S2. HR-ESI-MS spectrum of CRM A; Fig. S3.1H NMR spectrum of CRM A in DMSO-d6; Fig. S4.13C NMR spectrum of CRM A in DMSO-d6; Fig. S5. CRM A production comparison of different generations of mutants; Fig. S6. The quantitative HPLC standard curves. [file 12934_2020_1418_MOESM1_ESM.pdf]

Microbial Cell Factories

Activation and enhancement of Caerulomycin A biosynthesis in marine-derived *Actinoalloteichus* sp. AHMU CJ021 by combinatorial genome mining strategies

Yunchang Xie[1], Jiawen Chen[1], Bo Wang[3,4], Tai Chen[3,4], Junyu Chen[1], Yuan Zhang[2], Xiaoying Liu[2]*, Qi Chen[2]*

[1] Key Laboratory of Functional Small Organic Molecule Ministry of Education and Jiangxi's Key Laboratory of Green Chemistry, Key Laboratory of Protection and Utilization of Subtropic Plant Resources of Jiangxi Province, School of Life Sciences, Jiangxi Normal University, Nanchang 330022, China.

[2] School of Life Sciences, Anhui Medical University, Hefei 230032, China.

[3]Guangdong Provincial Key Laboratory of Genome Read and Write, Shenzhen Engineering Laboratory for Innovative Molecular Diagnostics, Guangdong Provincial Academician Workstation of BGI Synthetic Genomics, BGI-Shenzhen, Beishan Industrial Zone, Shenzhen, 518083, China.

[4] China National GeneBank, BGI-Shenzhen, Jinsha Road, Shenzhen, 518120, China.

*Correspondence: liuxiaoying@ahmu.edu.cn, chenqi@ahmu.edu.cn

Contents

Table S1	Genome features of *Actinoalloteichus* sp. AHMU CJ021	3
Table S2	Number of genes associated with the general COG functional categories	4
Table S3	The biosynthetic gene clusters of secondary metabolites in *Actinoalloteichus* sp. AHMU CJ021 analyzed by antismash 5.0	5
Table S4	Deduced functions of the open reading frames (ORFs) indicated in Fig. 2	6-7
Table S5	Fermentation media used for caerulomycin A (CRM A) production	8
Table S6	The mutants obtained from ribosome engineering experiments	9
Table S7	The CRM A production comparison of three *camE*-expressing mutants	9
Table S8	^{1}H NMR (500 MHz) and ^{13}C NMR (125 MHz) spectral data of CRM A in DMSO-d_6	10
Table S9	The comparison of selected mutants generated from UV mutagenesis	10
Table S10	CRM A production titer of optimal mutant XC-11GUR	10
Table S11	The dose of all factors in medium N2 by using Plackett-Burman Design	11
Table S12	Screening of significant variables for CRM A production in Medium N2 by using Plackett-Burman Design	12
Table S13	The effects of all factors of Medium N2 for CRM A production by using Plackett-Burman Design	12
Table S14	The dose of important factors in response surface analysis	12
Table S15	The design of experiments and response of CRM A production	13
Table S16	The primers used in identification of gentamycin-resistant mutant	13
Table S17	The primers used in gene expression analysis	13
Fig. S1	Phylogenetic tree of *Actinoalloteichus* sp. AHMU CJ021	14
Fig. S2	HR-ESI-MS spectrum of CRM A	15
Fig. S3	^{1}H NMR (500 MHz) spectrum of CRM A in DMSO-d_6	16
Fig. S4	^{13}C NMR (125 MHz) spectrum of CRM A in DMSO-d_6	17
Fig. S5	CRM A production comparison of different generations of mutants	18
Fig. S6	The quantitative HPLC standard curves	19
References		20

Table S1 Genome features of *Actinoalloteichus* sp. AHMU CJ021

Features	Chromosome
Genome topology	linear
Genome size (bp)	6,825,770
G+C content (%)	72.31
Total number of genes	5,389
Protein-coding genes	5,067
rRNA genes	9
tRNA genes	53
Number of putative gene clusters	22

Table S2 Number of genes associated with the general COG functional categories

Code	% age	Description
A	0.04	RNA processing and modification
B	0.08	Chromatin structure and dynamics
J	6.48	Translation, ribosomal structure and biogenesis
K	6.74	Transcription
L	6.02	Replication, recombination and repair
D	0.84	Cell cycle control, cell division, chromosome partitioning
M	4.08	Cell wall/membrane/envelope biogenesis
N	0	Cell motility
O	3.75	Posttranslational modification, protein turnover, chaperones
T	3.45	Signal transduction mechanisms
U	0.72	Intracellular trafficking, secretion, and vesicular transport
V	3.41	Defense mechanisms
W	0	Extracellular structures
Y	0	Nuclear structure
Z	0	Cytoskeleton
C	7.75	Energy production and conversion
E	11.12	Amino acid transport and metabolism
F	2.99	Nucleotide transport and metabolism
G	7.28	Carbohydrate transport and metabolism
H	3.92	Coenzyme transport and metabolism
I	4.8	Lipid transport and metabolism
P	7.16	Inorganic ion transport and metabolism
Q	5.43	Secondary metabolites biosynthesis, transport and catabolism
R	7.83	General function prediction only
S	6.11	Function unknown

Table S3 The biosynthetic gene clusters of secondary metabolites in *Actinoalloteichus* sp. AHMU CJ021 analyzed by antismash 5.0

Cluster	Type	From	To	Most similar known biosynthetic gene cluster (percent of similarity)
1	Thiopeptide	84761	120763	Nocathiacin (29%)
2	T1PKS	134077	220778	Butyrolactol A (66%)
3	T1PKS-NRPS	239441	386762	Caerulomycin A (95%)
4	Ladderane	429171	471438	Vancosamine (100%)
5	Lantipeptide	577163	610472	-
6	T2PKS	634691	707296	Arixanthomycin (22%)
7	Lantipeptide	833022	853627	-
8	Siderophore	917838	930581	Ficellomycin (9%)
9	Bacteriocin	955078	964882	-
10	Ectoine	2196292	2206726	Ectoine (100%)
11	NRPS	3930128	4107440	Surugamide (19%)
12	Terpene	4287071	4305038	Caerulomycin A (8%)
13	Other KS	4471065	4515508	-
14	Terpene	5248817	5267265	SF2575 (6%)
15	T1PKS-NRPS	5337375	5438577	SGR-PTMs (66%)
16	NRPS	5458085	5498993	Streptophenazine (7%)
17	NRPS	5505765	5559044	Nocardicin A (85%)
18	NRPS	5596321	5656214	Desotamide (9%)
19	NRPS-Siderophore-Terpene	5662296	5777479	Carotenoid (45%)
20	Bacteriocin	5885174	5895608	--
21	NRPS	5950404	5992637	Malonomycin (11%)
22	Lantipeptide-T1PKS-NRPS	6067690	6733574	Apoptolidin (43%)

Table S4 Deduced functions of the open reading frames indicated in **Fig. 2**, and its comparison with two other CRM A biosynthesis gene cluster **(**with accession number of JF419316 and JQ687072.1)

Gene	Size a	Proposed function	crm cluster	Size a	Accession number	cae cluster	Size a	Accession number	Identity/Similarity (%)
orf(-3)	1099	Putative formate acetyltransferase	*orf(-3)*	1099	AFD30968.1				99/99
orf(-2)	40	50S ribosomal protein	*orf(-2)*	40	AFD30967.1				100/100
orf(-1)	269	aklanonic acid methyltransferase	*orf(-1)*	269	AFD30966.1	*caeG2*	275	AFK24505.1	100/100
camT1	430	putative transporter	*crmT1*	430	AFD30965.1	*caeH3*	430	AFK24506.1	99/100
camR1	183	putative transcriptional regulator	*crmR1*	183	AFD30964.1	*caeI2*	211	AFK24507.1	99/100
camT2	608	putative ABC transporter	*crmT2*	608	AFD30963.1	*caeH1*	608	AFK24508.1	99/99
camT3	581	putative ABC transporter	*crmT3*	581	AFD30962.1	*caeH2*	581	AFK24509.1	99/99
camH	407	isobutylamine *N*-hydroxylase	*crmH*	407	AFD30961.1	*caeB5*	407	AFK24510.1	100/100
camX	**111**	unknown	--	--	--	--	--		--
camM	353	putative O-methyltransferase	*crmM*	353	AFD30960.1	*caeG1*	353	AFK24511.1	100/100
camG	523	putative aminotransferase	*crmG*	523	AFD30959.1	*caeC*	523	AFK24504.1	99/100
camF	371	FAD-binding monooxygenase	*crmF*	371	AFD30958.1	*caeB6*	371	AFK24512.1	99/100
camE	650	2, 3-dihydroxybenzoate-AMP ligase	*crmE*	650	AFD30957.1	*caeA1*	650	AFK24513.1	99/100
camC	416	L-Lysine 2-aminotransferase	*crmC*	416	AFD30956.1	*caeP1*	416	AFK24514.1	100/100
camD	395	monomeric sarcosine oxidase	*crmD*	395	AFD30955.1	*caeP2*	395	AFK24515.1	99/100
camA	2484	non-ribosomal peptide synthetase/polyketide synthase	*crmA*	2484	AFD30954.1	*caeA2*	2484	AFK24516.1	99/99
camB	1050	non-ribosomal peptide synthetase	*crmB*	1050	AFD30953.1	*caeA3*	1047	AFK24517.1	99/99
camI	391	acyl-CoA dehydrogenase	*crmI*	391	AFD30952.1	*caeB1*	386	AFK24518.1	99/100
camJ	234	putative type II thioesterase	*crmJ*	234	AFD30951.1	*caeA4*	222	AFK24519.1	99/100
camR2	183	putative regulator	*crmR2*	183	AFD30950.1	*caeI1*	176	AFK24520.1	99/99
camL	421	amidohydrolase	*crmL*	421	AFD30949.1	*caeD*	421	AFK24521.1	100/100

camN	369	GriC-like dehydrogenase component	*crmN*	369	AFD30948.1	*caeF*	396	AFK24522.1	99/100
camO	466	GriD-like dehydrogenase component	*crmO*	466	AFD30947.1	*caeB2*	456	AFK24523.1	100/100
camK	500	secreted FAD-binding protein	*crmK*	500	AFD30946.1	*caeB3*	533	AFK24524.1	99/99
orf1	150	F420-dependent NADP oxidoreductase coenzyme	*orf1*	205	AFD30945.1	*caeB4*	211	AFK24525.1	100/100
orf2	127	Transcriptional regulator	--			*caeI3*	127	AFK24526.1	
orf3	242	Monooxygenase				*caeB7*	242	AFK24527.1	

Table S5 Fermentation media used for caerulomycin A (CRM A) production

**Strain**	Culture conditions	
	Production medium	Time[b]
Streptomyces caerulezls PRL 1687 [(1)]	**Medium 1**: starch-Czapel medium: soluble starch 20 g/L, K_2HPO_4 1 g/L, $MgSO_4 \cdot 7H_2O$ 0.5 g/L, KCl 0.5 g/L, $NaNO_3$ 2 g/L, $CaCO_3$ 2 g/L, sea salt 30 g/L[d], pH 8.0, 30 °C;	7
Actinoalloteichus cyanogriseus WH1-2216-6[(2)]	**Medium 2**: glucose 20 g/L, beef extract 3 g/L, yeast extract 10 g/L, soluble starch 10 g/L, peptone 10 g/L, K_2HPO_4 0.5 g/L, $MgSO_4$ 0.5 g/L, $CaCO_3$ 2 g/L, and marinum salt 33 g/L, pH 7.0, 28 °C;	11
Actinoalloteichus cyanogriseus NRRL B-2194 [(3)a]	**Medium 3**: soluble starch 20 g/L, $NaNO_3$ 2 g/L, $CaCO_3$ 2 g/L, K_2HPO_4 1 g/L, KCl 0.5 g/L, $MgSO_4 \cdot 7H_2O$ 0.5 g/L, sea salt 30 g/L[d] pH 8.9, 28 °C;	4
	**Medium 4**: TSB medium (seed medium) + sea salt 30 g/L[d], 30 °C;	3
Actinoalloteichus cyanogriseus PM0525875[(4)a]	**Medium 5**: ASW-36P(1) medium + sea salt 30 g/L[d], 30 °C;	4
	**Medium 6**: ASW-274(1) medium (seed medium) + sea salt 30 g/L[d], 30 °C;	4
Actinoalloteichus sp. AHMU CJ021 (this study)	**Medium 7**: ISP2 medium + sea salt 30 g/L[d], 28 °C;	7
	**Medium N1**: soluble starch 20 g/L, glucose 5 g/L, yeast extract 2 g/L, peptone 2 g/L, $CaCO_3$ 2 g/L, K_2HPO_4 1 g/L, KCl 0.5 g/L, $MgSO_4 \cdot 7H_2O$ 0.5 g/L, sea salt 30 g/L, pH 7.5, 28 °C;	7
	**Medium N2**: soluble starch 20 g/L, yeast extract 2 g/L, peptone 2 g/L, $CaCO_3$ 2 g/L, $(NH_4)_2SO_4$ 2 g/L, K_2HPO_4 1 g/L, $MgCl_2$ 0.5 g/L, NaCl 1 g/L, sea salt 30 g/L, pH 7.5, 28 °C;	7
	**Optimized Medium N2**: soluble starch 10.67 g/L, yeast extract 3.83 g/L, peptone 2 g/L, $CaCO_3$ 2 g/L, $(NH_4)_2SO_4$ 1 g/L, K_2HPO_4 1 g/L, $MgCl_2$ 0.5 g/L, NaCl 1 g/L, sea salt 30 g/L, pH 7.5, 28 °C;	7

a: The fermentation process including two stages;

b: Number indicates the days;

c: The fermentation process was operated in ferment;

d: The sea salt was supplied into the original medium for this study;

e: The red color indicates the optimized ingredients

Table S6 The mutants obtained from ribosome engineering experiments

Name	Antibiotics resistance analysis		
	Type	Concentration (μg/mL)	MIC ratio level[a]
XC-1S	streptomycin (Str)	50	1
XC-2S	streptomycin (Str)	50	1
XC-3S	streptomycin (Str)	100	2
XC-4S	streptomycin (Str)	50	1
XC-5S	streptomycin (Str)	150	3
XC-6S	streptomycin (Str)	150	3
XC-7S	streptomycin (Str)	200	4
XC-8S	streptomycin (Str)	300	6
XC-9S	streptomycin (Str)	200	4
XC-10G	gentamycin (Gen)	30	2
XC-11G	gentamycin (Gen)	30	2
XC-12G	gentamycin (Gen)	30	2
XC-13G	gentamycin (Gen)	15	1
XC-14G	gentamycin (Gen)	15	1
XC-15R	rifamycin (Rif)	20	2
XC-16R	rifamycin (Rif)	50	5
XC-17R	rifamycin (Rif)	100	10

a: MIC ratio level was the ratio of mutant antibiotic resistant concentration to strain's antibiotic minimal inhibition concentration;

b: Red color labeled strains were CRM A production mutants

Table S7 The CRM A production comparison of three *camE*-expressing mutants

Name	*camE* expressing level[a]	CRM A titer (mg/L)
XC-11G	5.35 ± 0.11	42.51 ± 4.22
XC-14G	2.13 ± 0.11	31.43 ± 2.51
XC-16R	0.91 ± 0.04	9.11 ± 0.37

a: gene expression comparison reference was the expression of 16S rDNA

Table S8. ^{1}H NMR (500 MHz) and ^{13}C NMR (125 MHz) spectral data of CRM A in DMSO-d_6

caerulomycin A		
Pos.	δ_C	δ_H, multi. (J/Hz)
1		
2	156.8, C	
3	106.4, CH	7.90, d (2.0)
4	166.5, C	
5	105.5, CH	7.32, d (2.0)
6	153.3, C	
7	148.7, CH	8.15, s
8(4-OMe)	55.5, CH_3	3.94, s
	-N-OH	11.73, s
1'		
2'	154.4, C	
3'	120.7, CH	8.38, d (7.0)
4'	137.2, CH	7. 94, dd (7.5, 7.0)
5'	124.4, CH	7.45, dd (7.5, 7.0)
6'	149.1, CH	8.68, d (7.5)

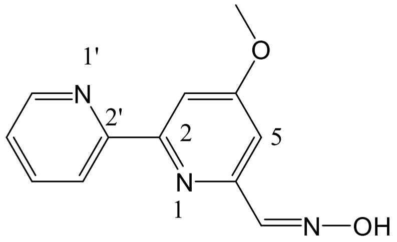

caerulomycin A (**1**)

Table S9 The comparison of selected mutants generated from UV mutagenesis

Strain	Inhibition Zone (mm)	CRM A titer (mg/L)
XC-11GU	25-27	78. 62 ± 3.55
XC-11GU-1	23-24	51.14 ± 2.77
XC-11GU-2	30-35	40.14 ± 1.89
XC-11GU-3	24-28	41.22 ± 1.18

Table S10 CRM A production titer of optimal mutant XC-11GUR

Medium[a]	CRM A titer (mg/L)	Fold level	Reference
Medium 1	160.54 ± 1.52	1.5	(1)
Medium 2	143.54 ± 2.99	1.7	(2)
Medium 3	168.15 ± 2.85	1.4	(3)
Medium 4	31.50 ± 1.11	7.6	TSB medium
Medium 5	131.42 ± 2.48	1.8	(4)
Medium 6	180.79± 2.16	1.3	(4)
Medium 7	113.91 ± 7.58	2.1	ISP2 medium
Medium N1	191.22 ± 1.11	1.2	This study
Medium N2	238.65 ± 3.14		This study

a: The detail composition of all nine media were described in Table S1

Table S11 The dose of all factors of medium N2 by using Plackett-Burman Design

Runs	Variable Level												CRM A
	X_1	X_2	X_3	X_{dv1}	X_4	X_5	X_6	X_{dv2}	X_7	X_8	X_9	X_{dv3}	Titer (mg/L)
**1**	1	1	-1	1	-1	-1	-1	-1	-1	1	1	1	441.22 ± 7.29
**2**	1	1	-1	-1	1	1	-1	1	-1	-1	1	-1	433.42 ± 8.11
**3**	-1	1	1	-1	-1	1	1	1	1	-1	1	1	390.91 ± 6.22
**4**	1	1	1	1	-1	-1	1	1	1	1	-1	-1	175.51 ± 2.11
**5**	-1	1	1	1	-1	1	1	-1	-1	-1	-1	-1	418.21 ± 5.88
**6**	-1	1	-1	1	1	1	-1	1	1	-1	-1	1	349.61 ± 5.13
**7**	-1	1	-1	-1	1	-1	1	1	-1	1	1	-1	539.42 ± 9.83
**8**	-1	-1	1	-1	1	-1	1	1	-1	1	-1	1	281.31 ± 3.44
**9**	1	1	1	1	1	-1	1	-1	-1	-1	1	1	255.41 ± 2.84
**10**	-1	-1	-1	1	-1	-1	-1	1	1	1	1	1	428.82 ± 8.13
**11**	1	-1	-1	-1	1	-1	1	-1	1	-1	-1	1	194.71 ± 2.99
**12**	1	1	1	-1	1	1	-1	-1	1	1	1	1	151.51 ± 1.13
**13**	-1	1	1	1	1	-1	-1	-1	1	-1	-1	-1	589.47 ± 10.22
**14**	1	-1	-1	1	-1	1	1	1	-1	-1	-1	1	160.91 ± 2.61
**15**	-1	1	1	-1	-1	1	-1	-1	-1	1	-1	1	364.51 ± 3.16
**16**	1	-1	1	1	1	1	-1	1	-1	1	-1	-1	127.01 ± 1.57
**17**	1	1	-1	-1	-1	1	1	-1	1	1	-1	-1	144.51 ± 1.78
**18**	-1	-1	-1	1	1	1	1	-1	1	1	1	-1	304.42 ± 6.98
**19**	-1	-1	-1	-1	-1	-1	-1	-1	-1	-1	-1	-1	498.07 ± 9.03
**20**	1	-1	1	-1	-1	-1	-1	1	1	-1	1	-1	165.01 ± 1.88

Table S12 Screening of significant variables for CRM A production in Medium N2 by using Plackett-Burman Design

Factor	Basic level	Low level (-1)	High level (+1)
X_1 /soluable strach	15 g/L	12 g/L	18 g/L
X_2 /yeast extract	2 g/L	1.5 g/L	2.5 g/L
X_3 /peptone	2 g/L	1.5 g/L	2.5 g/L
X_4/$CaCO_3$	2 g/L	1.5 g/L	2.5 g/L
X_5/$(NH_4)_2SO_4$	2 g/L	1.5 g/L	2.5 g/L
X_6/K_2HPO_4	1 g/L	0.8 g/L	1.2 g/L
X_7/$MgCl_2$	0.5 g/L	0.4 g/L	0.6 g/L
X_8 /NaCl	1 g/L	0.8 g/L	1.2 g/L
X_9/sea salt	30 g/L	25 g/L	35 g/L

Table S13 The effects of all factors in medium N2 for CRM A production by using Plackett-Burman Design

Factor	Effect	Coefficient	*t* Value	*p* Value
X_1 /soluble starch	-196.4	-98.2	-6.33	0.001
X_2 /yeast extract	109.7	54.9	3.19	0.015
X_3 /peptone	-74.8	-37.4	-2.34	0.052
X_{dv1} /dummy variable 1	13.5	6.8	0.44	0.676
X_4 /$CaCO_3$	-1.0	-0.5	-0.03	0.976
X_5 /$(NH_4)_2SO_4$	-89.5	-44.8	-2.80	0.026
X_6 /K_2HPO_4	-63.5	-31.8	-2.05	0.081
X_{dv2} /dummy variable 2	-13.9	-6.9	-0.43	0.677
X_7/$MgCl_2$	-67.3	-33.7	-2.17	0.067
X_9/NaCl	-54.6	-27.3	-1.76	0.122
X_9 /sea salt	48.2	24.1	1.44	0.193
X_{dv3}/dummy variable 3	-42.4	-21.2	-1.37	0.214

Table S14 The dose of important factors in response surface analysis

Level	X_1 /soluble starch	X_2 /yeast extract	X_5 /$(NH_4)_2SO_4$
-1	9 g/L	3 g/L	0.9 g/L
0	12 g/L	3.5 g/L	1.2 g/L
1	15 g/L	4 g/L	1.5 g/L

Table S15 The design of experiments and response of CRM A production

Runs	X_1 soluble starch	X_2 yeast extract	X_5 $(NH_4)_2SO_4$	CRM A Titer (mg/L)
1	-1	0	-1	590.91 ± 11.32
2	-1	0	1	515.63 ± 5.11
3	1	0	-1	481.03 ± 7.44
4	1	0	1	322.96 ± 6.16
5	0	-1	1	352.21 ± 3.28
6	0	1	1	491.33 ± 5.38
7	0	-1	-1	459.55 ± 3.12
8	0	1	-1	619.05 ± 10.14
9	1	1	0	439.95 ± 3.21
10	-1	-1	0	472.34 ± 7.81
11	1	-1	0	305.04 ± 6.47
12	-1	1	0	610.85 ± 3.88
13	0	0	0	574.91 ± 9.15
14	0	0	0	582.93 ± 10.22
15	0	0	0	560.32 ± 8.39

Table S16. The primers used in identification of gentamycin-resistant mutant

Sequence (5'-3')	Amplified products
**rps12-Fr**:ATGCCCACGATCCAGCAGCTG	30S ribosomal protein S12
**rps12-Re**:TTAGCTCTTCTCCTTCTTCGC	
**rpl6-Fr**:ATGTCGCGCATTGGGAAGCTG	50S ribosomal protein L6
**rpl6-Re**:TCACTTACCCGTCTTTCCGAC	
**16s-Fr**:AGAGTTTGATCCTGGCTCAGG	16S rDNA
**16s-Re**:AGGTGATCCAGCCGCACCTTC	

Table S17 The primers used in gene expression analysis.

Gene	Name	Sequence (5'-3')
camE	EPcamE-Fr	CGAACCGGAACAGGCCTTCG
	EPcamE-Re	CGTAGCACTGCTGGACCTGG
16S rDNA	EP16S-Fr	CTAACTACGTGCCAGCAGCC
	EP16S-Re	CTGATATCTGCGCATTCCAC

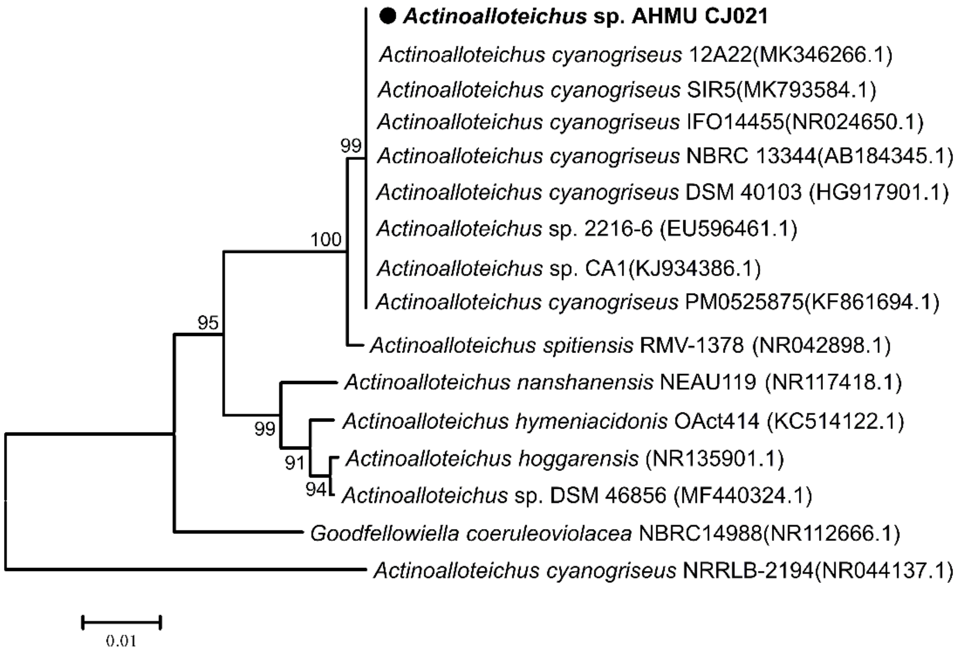

Fig. S1 Phylogenetic tree of *Actinoalloteichus* sp. AHMU CJ021. Numbers at the nodes represent bootstrap percentages obtained from 1000 replicates. The scale bar (0.01) represents nucleotide substitutions per site. Sequence alignment was performed with ClustalW. The tree was constructed with MEGA 7.0 software using neighbor-joining algorithm based on 16S rRNA gene sequences

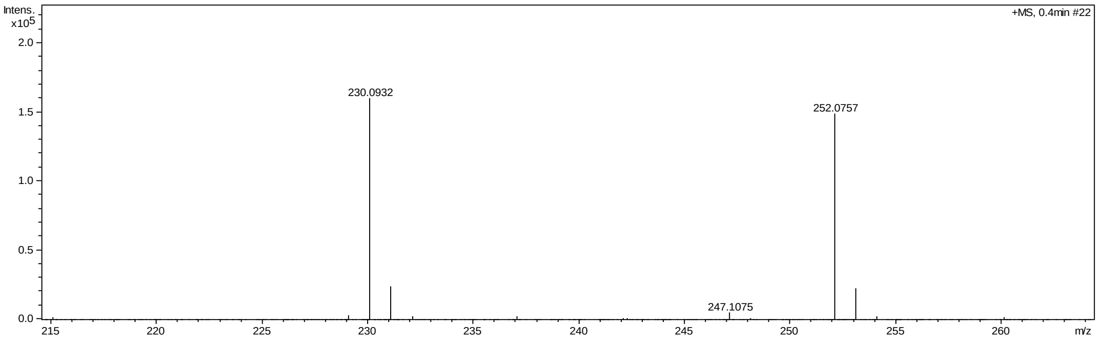

Fig. S2 HR-ESI-MS spectrum of CRM A

HR-ESI-MS(+) *m/z* $[M+H]^+$=230.0932 (cacld for $C_{12}H_{12}N_3O_2$, 230.0924)

HR-ESI-MS(+) *m/z* $[M+Na]^+$=252.0757 (cacld for $C_{12}H_{11}N_3NaO_2$, 252.0743)

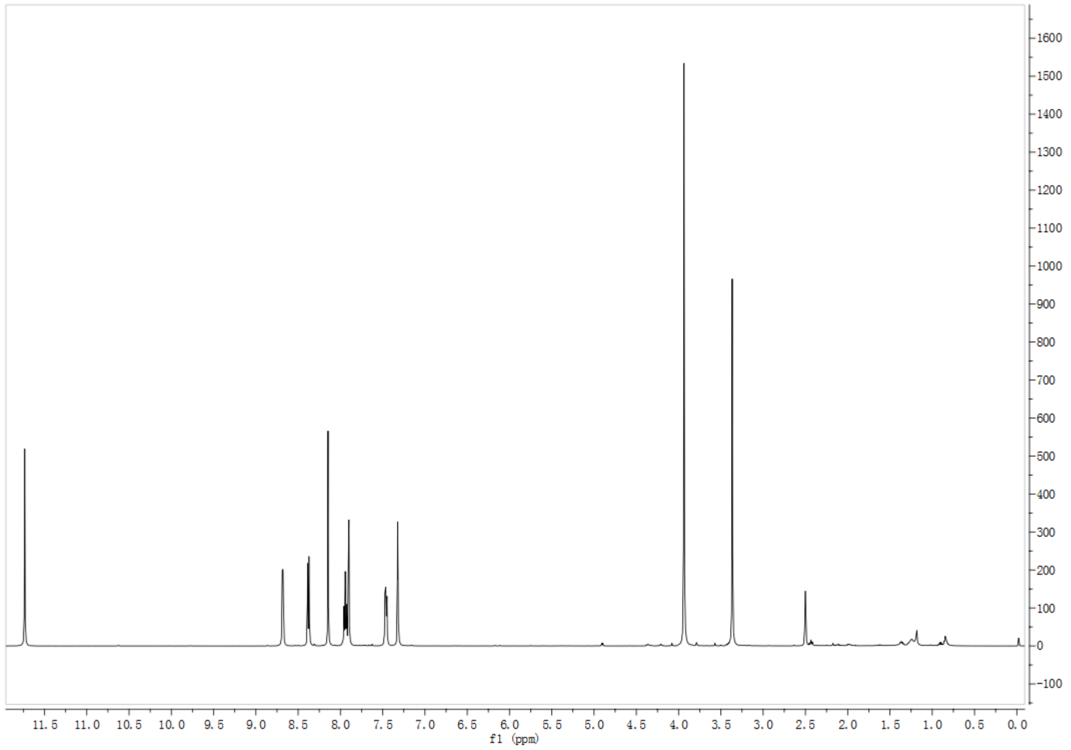

Fig. S3 ^{1}H NMR (500 MHz) spectrum of CRM A in DMSO-d_6

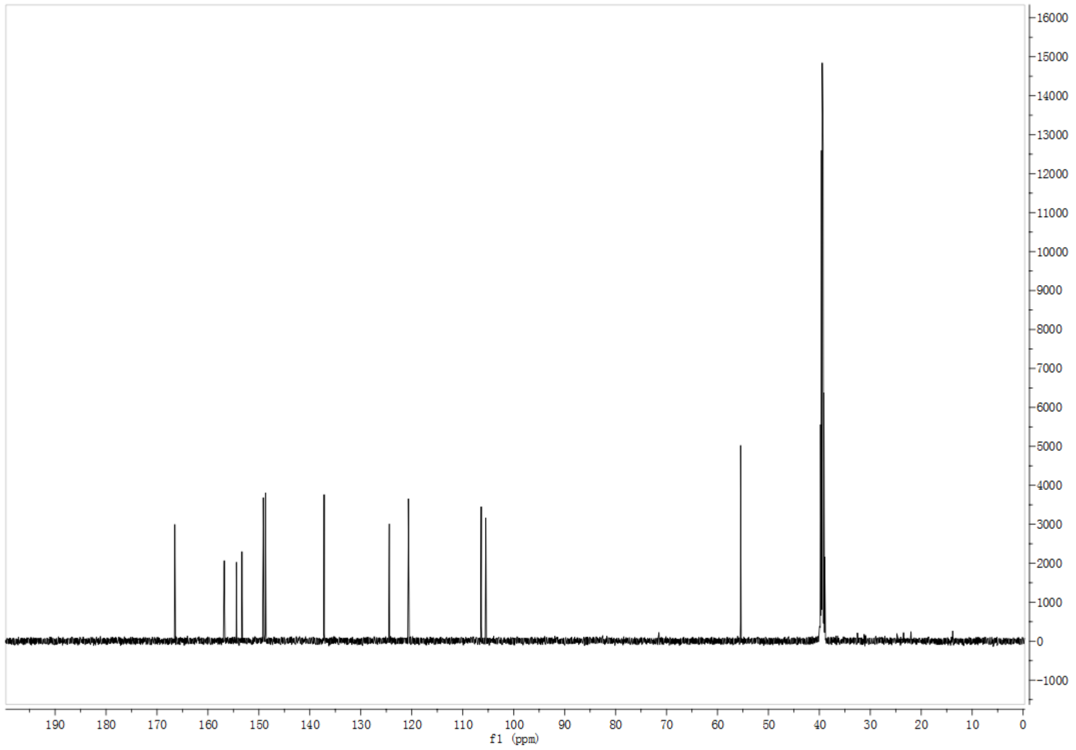

Fig. S4 ^{13}C NMR (125 MHz) spectrum of CRM A in DMSO-d_6

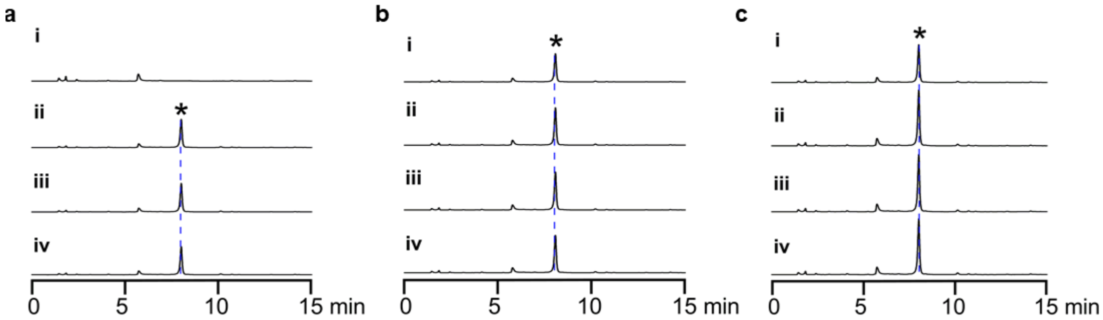

Fig. S5 CRM A production (asterisk) comparison of different generations of mutants. (**a**) i: wild type, ii-iv: first, second and third-generation strains of XC-11G, respectively. (**b**) i: XC-11G, ii-iv: first, second and third-generation strains of XC-11GU, respectively. (**c**) i: XC-11GU, ii-iv: first, second and third-generation strains of XC-11GUR, respectively

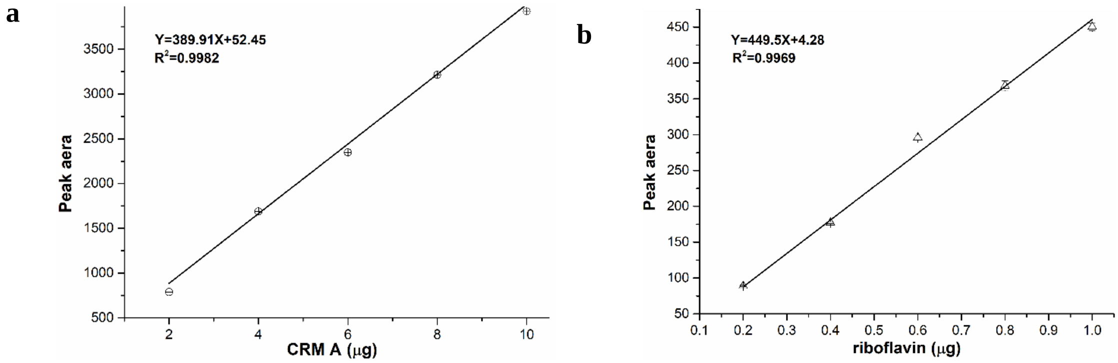

Fig. S6 The quantitative HPLC standard curve for titer calculation of CRM A (**a**) and riboflavin (**b**)

References

(1) Funk A, Divekar PV. Caerulomycin, a new antibiotic from *Streptomyces caeruleus* Baldacci. I. production, isolation, assay, and biological properties. Can J Microbiol. 1959; 5: 317–321.

(2) Fu P, Wang S, Hong K, Li X, Liu P, Wang Y, Zhu W. Cytotoxic bipyridines from the marine-derived actinomycete *Actinoalloteichus cyanogriseus* WH1-2216-6. J Nat Prod. 2011; 74(8): 1751–1756.

(3) Qu XD, Pang B, Zhang ZC, Chen M, Wu ZH, Zhao QF, Zhang QL, Wang YY, Liu Y, Liu W. Caerulomycins and collismycins share a common paradigm for 2, 2'-bipyridine biosynthesis via an unusual hybrid polyketide-peptide assembly logic. J Am Chem Soc. 2012; 134(22): 9038–9041.

(4) Ambavane V, Tokdar P, Parab R, Sreekumar ES, Mahajan GB, Mishra PD, D'Souza L, Ranadive P. Caerulomycin A-an antifungal compound isolated from marine actinomycetes. Adv Microbiol. 2014; 4(9): 567–578.
